# Artificial intelligence evaluation of nature based flood resilience in hilly terrain

**DOI:** 10.1038/s41598-025-19629-9

**Published:** 2025-10-10

**Authors:** Abdelkader Mabrouk, Inamullah Inam, Muhammad Zeeshan Qureshi, Tariq Ali, Nadir Murtaza, Mohamed Mohamed Ouda, Ahmed A. Alawi Al-Naghi, Dany Tasán Cruz

**Affiliations:** 1 Department of Civil Engineering, College of Engineering, Northern Border University, 73222 Arar, Saudi Arabia; 2 Department of Civil Engineering, Engineering Faculty, Laghman University, Mehtarlam, Afghanistan; 3 Department of Civil Engineering, University of Engineering and Technology, Taxila, Pakistan; 4 Department of Civil Engineering, Swedish College of Engineering and Technology, Wah, 47080 Pakistan; 5 Architecture Department, College of Architecture & Planning, King Khalid University, Abha, Saudi Arabia; 6 Civil Engineering Department, University of Ha’il, 55476 Ha’il, Saudi Arabia; 7 Universidad Nacional de Chimborazo, Riobamba, Ecuador; 8 Department of Civil Engineering, CECOS University of IT and Emerging Sciences, Peshawar, 25000, Pakistan

**Keywords:** Nature-based solution, Artificial intelligence, Flood resilience, SHAP analysis, Monte carlo simulation, Environmental sciences, Hydrology, Engineering

## Abstract

Hilly terrain has notable challenges of flash flooding, requiring a cost-effective and sustainable management approach. Nature-based solutions (NBS) provide significant sustainability, however need methodical assessment under diverse hydrological conditions. To fill this gap, the current investigation explores the significance of the NBS utilizing artificial intelligence (AI) techniques for optimizing flood resilience through the prediction of peak discharge generated from the hilly terrain. For this purpose, two AI advanced models including random forest (RF) and support vector regression (SVR) model were utilized for the assessment of laboratory scale data series of slope, rainfall intensity (P), and time ratio (T/Tc, T: total time, Tc: time of concentration) with flexible (FV) and rigid vegetation (RV). A total of 344 data series were collected, split into three different phases of training (70%), testing (15%), and validation (15%) for both AI models. The values of root mean square error (RMSE), coefficient of determination (R2), and mean absolute error (MAE), along with SHAP (SHapley Additive exPlanations) analysis, are used to assess the performance of AI models. Further Monte Carlo simulation was performed to clarify uncertainty and feature importance. The result demonstrated 8% more reduction of the peak discharge in the case of flexible vegetation, because of its surface resistance and infiltration capability. The RF model has greater prediction power compared to the SVR model Due to a higher R-value of 0.9809 for FV and 0.9906 for RV conditions. The result of SHAP analysis demonstrates greater influence of time ratio on peak discharge under FV (SHAP range:±25 and RV (SHAP range: ±30), while the moderate impact of rainfall intensity was observed in the case of FV (SHAP range: ±5) and RV (SHAP range: ±7). The findings concluded that AI-driven models utilized for NBS enhance resilience against flooding in hilly terrain. Urban planners and policymakers should utilize an AI-driven approach for nonlinear hydrological phenomena.

**Supplementary Information:**

The online version contains supplementary material available at 10.1038/s41598-025-19629-9.

## Introduction

Nature-based solutions (NBS) are widely accepted soft solutions for improving flood resilience due to their significant contribution. The higher infiltration capacity and a significant reduction in surface runoff of NBS have more effectiveness in mitigating flood risk^1,2^. According to the study conducted by Kibii et al.^3^, NBS reduces spontaneous water movement, along with the reduction of frequent worsening of environmental heat due to their permeable nature. Flash challenges such as climate change adaptation and flash flooding can be controlled by the utilization of the NBS^4^. Additionally, Raymond et al.^5^ observe that NBS need to be implemented within the context of structural systems that can adaptively respond to this practice by utilizing representatives from several fields to ensure the approaches are environmentally friendly and tailored to specific situations. Russo et al.^6,7^ show that integrated modern city planning tools to NBS may yield great improvements in the forecasting capability for flood control. The findings of these demonstrate that NBS is an indispensable component of a broader and multi-sectoral build for enhancing flash flood resilience.

Recent research has shown that flash floods can be handled not only through nature-based solutions but also have ongoing financial benefits. Although there is the initial high investment associated with NBS, these expenditures would be soft in comparison to those of conventional gray amenities, as its ongoing maintenance costs are much lower, rendering it a more sustainable financial solution, as stated by De-Brito and Evers^8^. This is a finding that agrees with the one from Liu and Zhang^9^, who proved that environmentally friendly structures such as wetlands, bioswales, or drainage ponds can reduce flash floods and at the same time improve public areas and the attractiveness of the city. Ferreira et al.^10^ emphasize the financial resilience that an NBS can provide to an affected area by mitigating the flood-related loss and, as a consequence, reducing the costs associated with the recovery. Kremer et al.^11^ further state that NBS provide extra benefits as well, such as additional air quality and richer biodiversity in cities for the benefit of the flash inhabitants’ overall well-being. Sharma et al.^12^ further found that NBS offer proportional, environmentally friendly, community, and financial advantages to cities beyond flood control, and cities have a greater long-term return on investment from NBS as compared with traditional infrastructure. The literature shows that NBS not only makes cities more resistant to floods, but also to the ongoing sustainability of cities and the financial stability of cities. NBS are natural buffers that help to reduce the effects of floods due to their ability to absorb and hold onto surplus water during periods when it is raining. This means that restoring these natural environments reduces the likelihood of flooding downstream, since these environments are better at controlling water flows. According to Debele et al.^13^ wetland types of NBS serve a major benefit over ordinary grey facilities, with the benefit of enhancing suffer quality, ecological diversity, as well as mitigating the effects of extreme floods. These two advantages make flood mitigation strategies involving the restoration of wetlands into an attractive option in flash and rural areas. Moreover, Ferreira et al.^14^ indicate that by restoring wetlands and incorporating them with ecologically designed, natural water absorption can be increased to minimize the risk in coastal areas. According to Debele et al.^13^ mangroves could protect fragile coasts by reducing wave heights by up to 60%. Burke and Spalding^15^ also demonstrate how coral reefs essentially act in the same way and do their part in dissipating wave energy before it lands on a coastline and does the damage of a storm or sea level rise. Apart from safeguarding marine wildlife, the rehabilitation of these coastal habitats also promotes human habitation. Rottmueller et al.^16^ suggest putting mangroves and coral reefs within the scope of coastal measures as a more environmentally friendly and cheaper option to build barriers or artificial sea walls and as a continuous solution to the growing risks of climate change.

More recently, AI approaches have been shown to have a great promise in flood forecasting and hazard mitigation, especially in the flash environment. Liu and Zhai^17^, for instance, examined how past rainfall data as well as the immediate time meteorological data can be foreseen through large datasets using machine learning such as artificial neural networks (ANN) and support vector machines (SVM). Because policymakers can take preventive steps, these AI models offer accurate and immediate warnings that can control the harm. Carlos et al.^18^ have emphasized the hybrid and combined use of AI with traditional gray infrastructure to enhance forms of flood resistance in metropolitan areas utilizing a multi-objective optimization model. Such a combined approach ensures that green and gray infrastructures will function together in their ability to absorb and react to flood occurrences in a city. In a recent paper, Liu et al.^19^ and Pham et al.^20^ use AI for the environmental network assessment to model the flood-prone regions. Increasingly, such ecological functions that control natural catastrophes are evaluated with geographical distribution and behavior of models driven by AI. Araújo et al.^21^ describe that machine learning techniques such as random forests (RF) and gradient boosting algorithms have been brought to support assessing how cities can use green infrastructure (such as forest areas and green roofs) to reduce the temperature of the heatwaves. The effects of upcoming droughts, for example, can be forecasted by these AI models, together with recommending the best locations for green infrastructure installation based on data on land use, plant cover, and microclimatic variables. Bae et al.^22^ also used AI-based calculation to investigate the long-term effects of environmentally friendly buildings in cities on their environments.’ In a framework of flood control, Gao et al.^23^ combined AI to improve green-grey solutions for infrastructure to improve the resilience of flash settings. Study by Liu et al.^17^ mentions that it is increasingly using convolutional neural networks (CNNs) and deep learning algorithms to forecast the simultaneous occurrence of various vulnerabilities in very flashized metropolitan regions. Bosch et al.^24^ investigated the effectiveness of AI in assessing green infrastructure scenarios with their combined effect on reducing the cumulative influence of heat waves and flash floods. Being able to forecast cascade effects, where a flood can trigger landslides in areas susceptible to landslides, this multi-hazard approach especially benefits cities threatened by seasonal hazards.

In the disciplines of urban planning and landscape architecture, developing effective flood risk management strategies for hilly urban areas requires a multifaceted approach that integrates both traditional and innovative methodologies. Historically, urban centres have relied on defensive measures such as dikes and levees, which, while effective in the short term, often neglected broader environmental impacts and long-term resilience^25–27^. Modern strategies emphasize the integration of blue-green infrastructure, which harmonizes urban development with natural water cycles, enhancing resilience and sustainability^27^. Effective flood management in urban areas necessitates a holistic approach that incorporates land-use planning, stormwater management, infrastructure design, and emergency response, ensuring that these elements are part of a comprehensive mitigation plan^28^. Architectural strategies, such as constructing buildings at elevations or suitable gradients, are crucial in fortifying structures against floods, especially in flood-prone areas^29^. Landscape architecture also plays a significant role, with nature-based solutions and green infrastructure being employed to mitigate flood risks and enhance resilience through interdisciplinary collaboration and community engagement^30^. The transition to adaptive flood risk management strategies, which focus on reducing the impacts of floods rather than solely preventing them, has shifted some responsibilities to non-state actors, including developers and residents, who are now more involved in adaptation efforts^31^. Urban flood risk management is further complicated by the increasing urbanization and climate change, which exacerbate flood risks by reducing natural catchments and increasing surface runoff^32,33^. Therefore, sustainable flood management requires a combination of structural and non-structural measures, including community involvement and the protection of natural river basins^33^. The integration of these diverse strategies, supported by dynamic flood modeling and a focus on socio-economic and environmental impacts, is essential for developing effective flood risk management plans in hilly urban areas^34,35^.

Nature-based solutions (NBS) have been reported as potential solutions to flood risk, especially in vulnerable regions that are rapidly urbanizing. Such solutions rely on ecologically beneficial processes, including infiltration, retention, and flow resistance caused by vegetation, to make the environment more resilient to floods and environmental rehabilitation^1^. Nevertheless, in the domain of reduction in peak runoff, biotic interventions, such as wetlands, bioswales, green roofs, and riparian buffers, and their role in maintaining hydrological balance within the city, many research studies have focused on their superiority in the last twenty years^10,12^; hence there is need to balance the positivity rhetoric with the arrival of artificial intelligence (AI) methods in the field, which have transformed the optimization and modeling of these interventions. Recent AI advances promote powerful methods to predict floods and estimate runoff, as well as analyze the sensitivity of features in hydrological systems that are both complex and nonlinear^9^. Due to the potential to detect complex patterns in the data, machine learning (ML) and deep learning algorithms (changing list to: Random Forest (RF), Support Vector Regression (SVR), Artificial Neural Network (ANN), and hybrid ones like ANN-PSO or ANFIS-SVR) are increasingly used in studies of various phenomena in hydrology^36,37^. As an example, RF and SVR have demonstrated a higher ability to model streamflow, rainfall-runoff relationships, and peak discharge forecasts under different terrain conditions^38^. Such methods are better than the conventional empirical and physics-based models as they fit high, non-linear data without a prior assumption. In addition, the recent combination of AI with such explainability tools as SHAP (SHapley Additive exPlanations), permutation importance, and Partial Dependence Plots (PDP) has greatly enhanced model interpretability further, empowering practitioners to understand which features have the greatest effect on the behavior of floods^39^.

Additionally, some researchers have investigated hybrid frameworks involving the hybrid coupling of AI models to hydrodynamic simulations or Geographic Information Systems (GIS) to increase the spatial recollection and precision of decision-making^23^. This has provided avenues to evaluate NBS performance in the conditions of climate change, land-use conversions, and vegetation processes. AI is also applicable in uncertainty quantification using Monte Carlo Simulations^40^, thereby having a potential application in resilience-based planning. Still, the literature lacks a study of merging the AI methods with the datasets of experimental scale, more specifically, assessing the influence of various vegetation patterns, i.e., rigid and flexible ones, on the peak discharge. This gap is addressed in the proposed study to propose an AI-based framework to predict peak discharge based on the laboratory-scale experiments under NBS conditions in terms of RF and SVR, and to understand the interaction of features with the help of explainable AI tools. The combination will be helpful in future research on how to optimize the flood resilience strategy and scale the AI-NBS framework to real-world use.

Climate change and flash floods cause variations in rainfall intensity and alter natural drainage systems, resulting in critical challenges such as flood resilience in hilly terrain^41,42^. However, for mitigating flash floods, previous studies reported the contribution of NBS, but their effectiveness was not explored under diverse hydrological conditions through a systematic analysis. Previous research lacks the investigation of optimized NBS for flood mitigation in hilly regions by developing an AI-based approach. Therefore, this paper fills this gap by developing optimized AI-based models for analysing the peak discharge generated from the hilly terrain under various types of vegetation. The objectives of the current research are to (1) investigation of flood mitigation on hilly terrain under varying rainfall intensities and slope utilizing vegetation (flexible vs. Rigid), (2) develop a high accuracy AI models like RF and SVR for the precise prediction of the peak discharge, and (3) assess the influence of the various input parameters on the prediction of the peak discharge through interpretable AI techniques like SHAP analysis, partial dependence plot, monte Carlo simulation, cumulative accuracy curve, and error distribution. This paper provide valuable insight on the integration of laboratory-scale experiment with AI models for optimization of NBS in hilly terrain, filling the existing gap in practical flood resilience and theoretical approach.

## Methodology

### General description

**Fig. 1:**
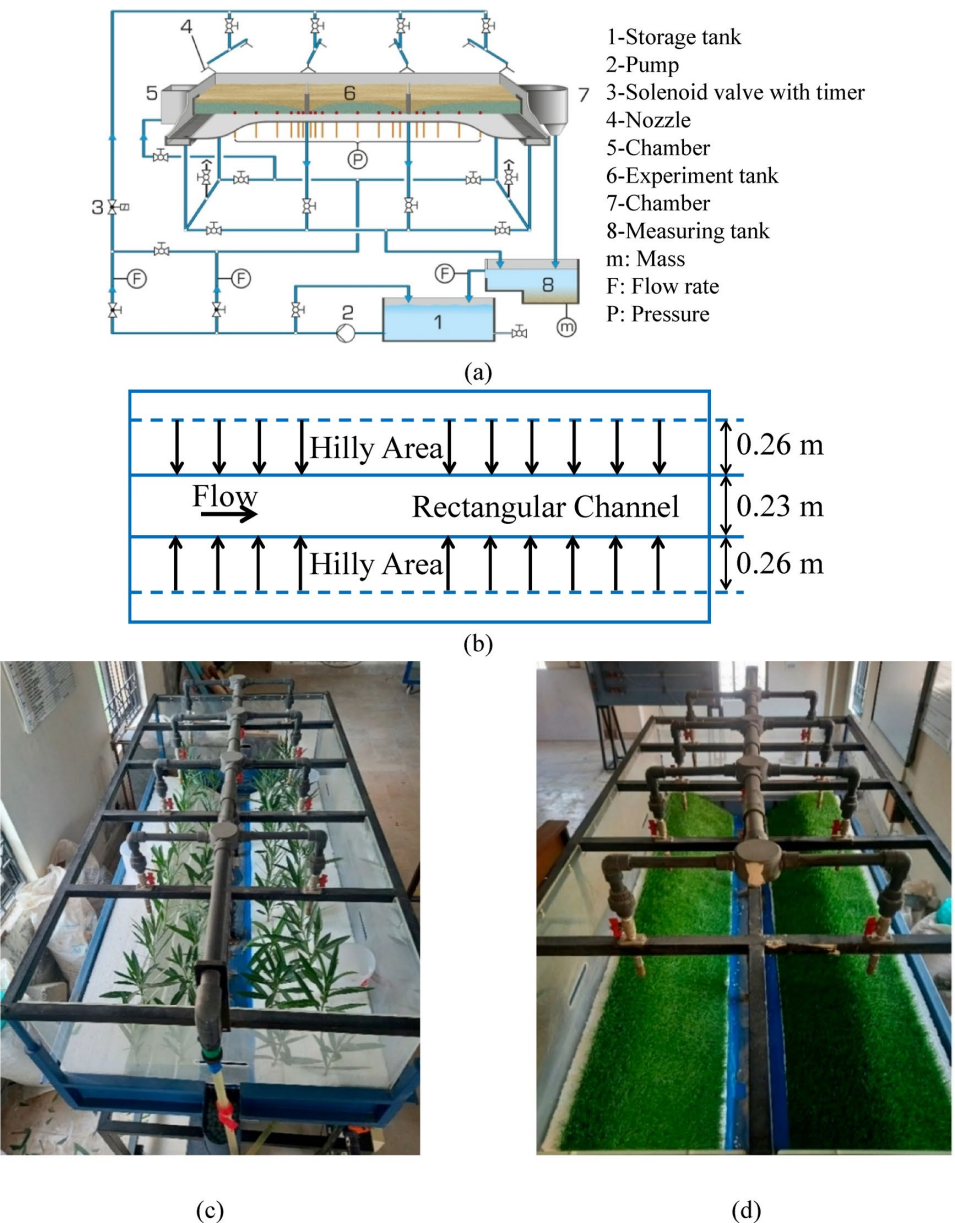
Experimental setup adopted by Rehman et al.^43^ for investigating peak discharge (**a**) rainfall simulator (**b**) schematic of top view (**c**) rigid vegetation conditions adopted in simulator (**d**) flexible vegetation conditions adopted in simulator.

The purpose of the current investigation was to predict peak discharge originating from the hilly terrain under diverse vegetation conditions, such as flexible and rigid vegetation utilizing artificial intelligence (AI) models. The primary goal was to investigate the impact of nature-based solutions in hilly terrain under different hydrological conditions. The experimental methodology of the current research was adopted by Rehman et al.^43^. They utilized laboratory-scale experiments through a rainfall simulator (FM-1849-45 by Infinit Technologies, Rosedale, MD, USA) (Fig. 1a) by simulating consistent rainfall under diverse conditions of terrain slope to investigate the impact of vegetation on the peak discharge (Q) as shown in Fig. 1a–d. The current research utilized advanced AI models including random forest (RF) and support vector regression (SVR) models through a dataset derived from the study conducted by Rehman et al.^43^. The dataset derived from the study conducted by Rehman et al.^43^ employed a rainfall simulator to generate Artificial rainfall over a predefined slope under diverse vegetation conditions. The slope of the terrain varied from 0° to 2° and rainfall intensities ranged from 0.3 cm/min to 0.5 cm/min respectively (Table 1). In this study, hilly terrain was selected considering the geomorphological and hydrological phenomenon rather than only based on the magnitudes of steep slope. In hydrological simulations, even shallow slopes (0–2) may model the hilly or undulated landscape when varied adequately in the laboratory scale, particularly during intense rainfall events, whereby accumulation of the flow and runoff behave in the same way as in real hillslope catchments^43^. The main aim of this paper was to study the impact of vegetation on overland flow behavior in shallow slope areas, where the initiation of flow, surface detention, and infiltration processes are very sensitive to surface resistance. In terms of rainfall intensity range (0.3–0.5 cm/min), the choice of this range of rainfall is due to flash flood similar rainfall rates measured in the tropics and subtropics, which is condensed into appropriate values for the laboratory^13^. These intensities create enough hydrological stress to be able to find peak discharge variation in various vegetative conditions, where repetition and control Are possible in the laboratory-scale setting, following earlier experimental studies of NBS by others. A total of 344 data series of the rainfall intensity, terrain slope, Time ratio (T/Tc, where T is the total time and Tc is the time of concentration), and peak discharge were derived for both flexible and rigid vegetation conditions from the study conducted by Rehman et al.^43^. The time noted from the start of the rainfall-runoff generated in a laboratory channel (experiments) from the initiation of rainfall to the flow of the runoff observed at the end of the channel is known as the total time. It includes the increasing and the decreasing portions of the hydrograph. They utilized various vegetation conditions, including no vegetation, flexible (artificial grass mats), rigid (tree branches), and a combination of both under different slope and rainfall intensities. However, the current study focused on utilizing AI models for the dataset of flexible and rigid vegetation conditions. For the development of the AI models, a dataset related to slope (%), rainfall intensity (P), and Time ratio (T/Tc) was considered as independent variables, while the peak discharge was considered as a dependent variable.

**Fig. 2:**
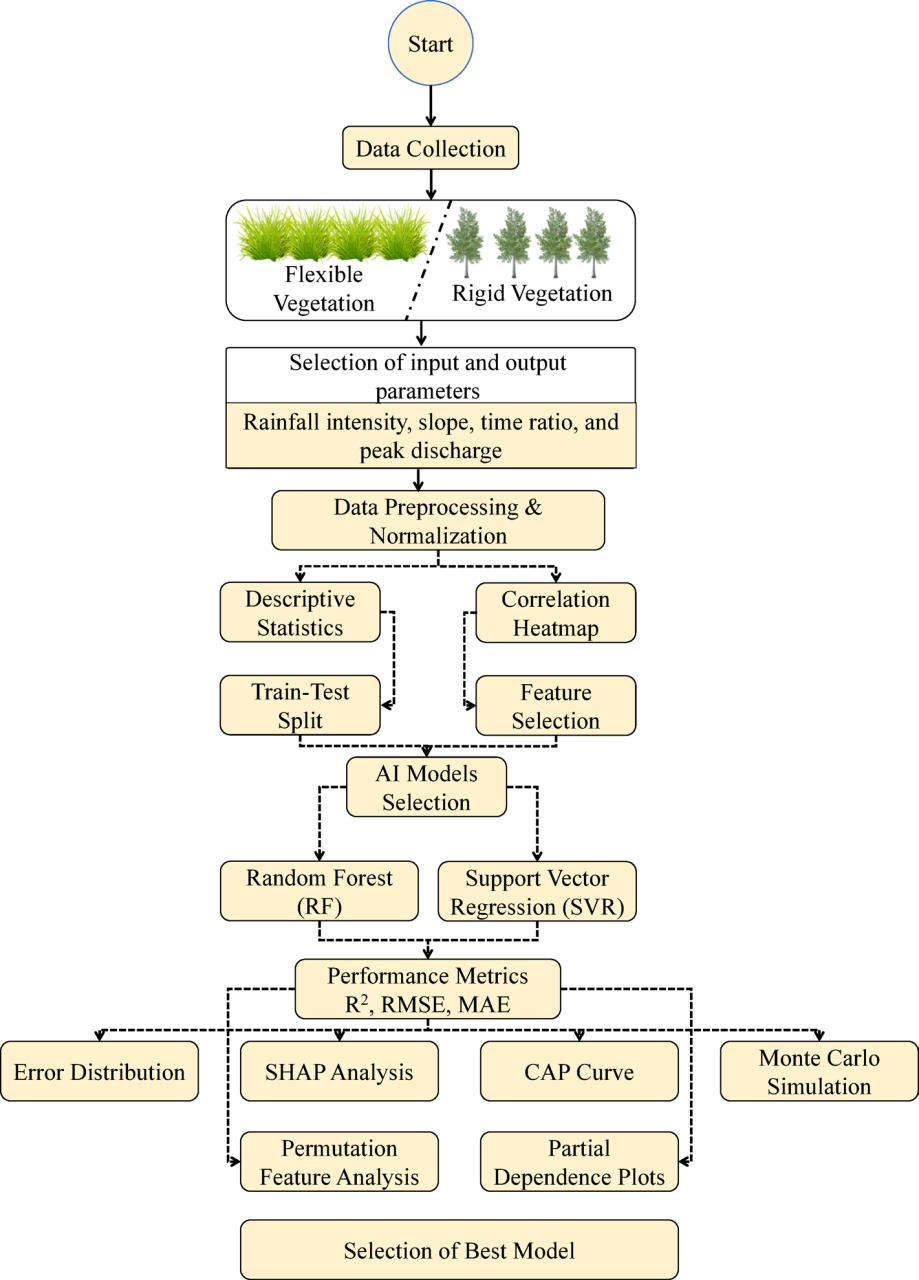
Flowchart of methodology adopted in the current research.

To ensure consistency and accuracy, a collected dataset was preprocessed. Within a dataset, units were standardized, and missing entities were removed to achieve a precise prediction of peak discharge. The dataset was split into three phases, namely training, testing, and validation phases, to develop an AI model. Training, testing, and validation datasets of 70%, 15%, and 15%, respectively, were assigned to the model, and the approach in Xu and Goodacre^44^ was followed. Such divisions offer unbiased model performance and the building of robust models. The relation between the input and the output parameters was understood before developing the AI model through a correlation heatmap. The evaluation of the multicollinearity and the selection of appropriate variables for model development is provided by this map. After generating the correlation heatmap, sophisticated AI models (e.g., RF and SVR) were chosen so that the peak discharge can be predicted accurately and with as high precision as possible from the invented artificial hilly terrain. RF model, which due to the handling problem of a nonlinear relationship between parameters, has been selected. This includes many trees, and the average values of peak discharge are obtained from predictions from each tree. Moreover, as stated by Bargam et al.^37^ the SVR model can also enable a high-dimensional regression function using a kernel function. The training of these RF and SVR models was done in MATLAB R2023b. After achieving the model, the performance indicators, which consisted of root mean square error (RMSE), mean absolute error (MAE), and coefficient of determination (R^2^), were assessed. The SHAP (Shapley Additive explanations) is developed for assessing the model predictive capability and each variable’s impact on the peak discharge. The study contributes to the individual parameters, such as time ratio (T/Tc), terrain slope, and rainfall intensity, to the predicted value of the peak discharge^39^. In addition, individual conditional expectation (ICE) curves of each model were plotted to observe the marginal impact of the individual factors on the peak discharge. Error distribution plots were used to evaluate the residual behavior as well as potential biases. Cumulative accuracy profile (CAP) curves are used to show how well the model performs, by non-equity measures, including peak discharge predictions. Descriptive statistics for both flexible and rigid vegetation are added in the subsequent section to help understand the reliability of the dataset. Figure 2 depicts the flowchart of the methodology adopted in the current research. The laboratory setup mainly focused on predicting the surface runoff generated from hilly terrain and the contribution of flexible and rigid types of vegetation in flow resistance. The limitations of the present paper are the exclusion of hydrological processes such as root-driven moisture regulation, transpiration, and interception. The exclusion of these processes limits the current AI-based framework utilized in this study to capture real-world scenarios of vegetation along with resisting flow. Further, the ecological complexity of the vegetation was also limited by replicating vegetation types (flexible and rigid) with the artificial grass mats and rigid branches. Therefore, it is recommended that future research should focus on the consideration of the hydrological processes interacting with vegetation for capturing the real-world significance of nature-based solutions.

Although previous research has made a significant contribution to predicting surface runoff by using an AI-based framework. However, the current study provides various novel contributions to existing literature. Previous studies focused on the prediction of runoff generated by considering a data series of different ungauged stations^45,46^. In contrast, the present paper focused on the systematic analysis of laboratory-scale experimental data series, including time ratio, slope variation, and rainfall intensity. Further, this study assessed the comparison of flexible and rigid vegetation in restricting runoff generated by using an advanced AI approach, which has not been adopted in the previous study^47^. However, a recently published work by Rezzoug et al.^48^ predicted flow resistance offered by rigid vegetation using an advanced AI-based framework. Therefore, the current study provides a novel framework of combining AI with nature-based solutions to urban or semi-urban flood-prone hilly regions. The current study not only relies on the utilization of AI techniques but also expands these techniques through SHAP analysis, which signifies the influence of each contributing variable in predicting output^39^. Previous studies mainly focused on the predictive capability of AI models while ignoring the interpretability^37^. The present paper combined interpretability analysis with partial dependence plots, permutation feature, and error distribution analysis to address this gap, providing robust behavior of the AI model in predicting runoff, critical for decision making. The variability of the probabilistic distribution and uncertainty have also been assessed by utilizing Monte Carlo Simulation, improving the significance of NBS under diverse terrain scenarios.

**Table 1:** Experimental and hydrological conditions adopted by Rehman et al.^43^.

Case ID	Vegetation type	Rainfall intensity (cm/min)			Channel slope	No’s of test
		P1	P2	P3		
1	FV	0.3	0.4	0.5	0⁰	3
2		0.3	0.4	0.5	1⁰	3
3		0.3	0.4	0.5	2⁰	3
4	RV	0.3	0.4	0.5	0⁰	3
5		0.3	0.4	0.5	1⁰	3
6		0.3	0.4	0.5	2⁰	3

### Code availability

In this study, different AI models have been utilized to observe the relationship between input and output parameters and the predictive values of the output parameter using different analyses such as correlation heatmap, model performance evaluation, SHAP analysis, cumulative accuracy profile, Monte Carlo simulation, and permutation feature importance. The custom code of each analysis is presented in the supplementary materials section of this paper.

### Descriptive statistics

In the current research, descriptive statistics were performed to understand the reliability of the data series collected in the case of flexible and rigid vegetation conditions utilized on hilly terrain, as summarized in Table 2. The analysis provides detailed comparisons of the flexible and rigid vegetation for mitigating runoff generated from the hilly terrain. The analysis describes each parameter, including input and output, for the effectiveness of the vegetation on hilly terrain. The descriptive statistics employed in this paper show the mean value of runoff generated of 18.01 L/min and 21.29 L/min in the case of flexible and rigid vegetation scenarios, respectively. The difference in their mean value was 22%, demonstrating the significance of the flexible vegetation in reducing runoff generation from the hilly terrain. Furthermore, the standard deviation of the rigid vegetation (21.29) was higher compared to flexible vegetation (18.01), showing a significant response of the flexible vegetation to the runoff generated. Flexible vegetation has significant resistance to the flow compared to the rigid vegetation scenario, demonstrated by the mean values of time ratio (flexible: 4.21 and rigid: 5.08). The consistency of the slope and rainfall reflected from their mean value of 1.03% and 7.05 across both types of vegetation. These measured outcomes confirm the result that flexible vegetation is better in delaying peak discharge when compared to rigid vegetation tested under diverse hydrological conditions. The result of the mean values for the peak discharge (runoff) and time ratio (T/Tc) shows that flexible vegetation has greater capability in delaying the peak discharge generated from the hilly terrain due to higher infiltration and resistance capability compared to rigid vegetation conditions. In contrast, rigid vegetation only resists discharge generated from the hilly terrain and has a minimum detention capacity that may allow faster surface flow. Similar to the mean value, it was observed that flexible vegetation provides greater values of the standard deviation, indicating its reflecting effect with rainfall conditions on hilly terrain. Upon comparing the results of the descriptive statistics, it was noted that flexible vegetation has greater performance in hydrological mechanisms because of its water retaining and reduction of peak discharge capacity compared to rigid vegetation conditions. Therefore, the result recommends the utilization of the flexible vegetation on hilly terrain for reducing surface runoff and flood risk. However, the influence of flexible vegetation on mitigating erosion risk should be considered in future research by incorporating soil infiltration rate and root depth variations.

**Table 2:** Summary of descriptive statistics. Where Q: discharge and t/tc: time ratio (T: total time and tc: time of concentration).

Parameter	Statistic	Flexible	Rigid
Rainfall intensity (P)	Mean	7.06	7.04
	Median	7	7
	Mode	8	8
	Standard deviation	0.82	0.82
Slope (%)	Mean	1.02	1.03
	Median	1	1
	Mode	2	2
	Standard deviation	0.82	0.81
T/Tc	Mean	5.08	4.21
	Median	3.74	3.38
	Mode	1	1
	Standard deviation	4.04	2.74
Q	Mean	25	32.12
	Median	20.83	30.3
	Mode	0	0
	Standard deviation	18.01	21.29

## Results and discussion

### Correlation heatmap

**Fig. 3:**
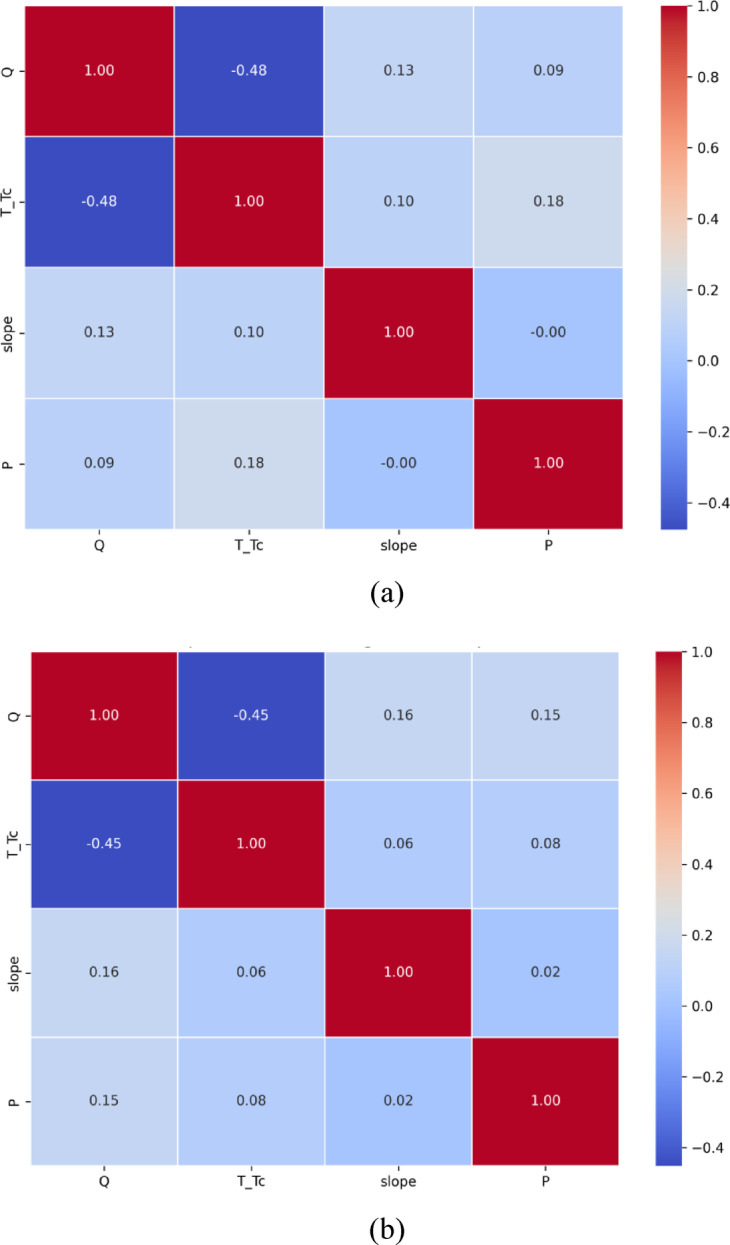
Correlation heatmap for developing a relationship between input and output parameters under flexible and rigid vegetation conditions (**a**) for flexible vegetation (**b**) for rigid vegetation. Where Q: peak discharge, T/Tc: time ratio, and P: rainfall intensity.

The relationship between the input and output parameters was analyzed for both types of vegetation, including flexible and rigid vegetation conditions, by using a correlation heatmap as shown in Fig. 3a, b. This map provides an understanding of the relationship between rainfall intensity, time ratio, terrain slope, and peak discharge under the impact of flexible vegetation on hilly regions. Analysis of the heatmap correlation shows a negative correlation between peak discharge and time ratio of *R* = −0.48, indicating that the peak discharge from the hilly terrain is reduced by increasing the time ratio and a similar trend was reported by Jahanshahi and Booij^49^, Due to the impact of climate and hydrological conditions on flood generation. This result shows the capability of the flexible vegetation on hilly terrain to minimize direct runoff and rates of peak runoff. Nevertheless, the slope correlation with an R value of 0.13 is somewhat higher than the time ratio correlation and the peak discharge. Using flexible vegetation conditions, the results show that terrain slope has a limited effect on peak discharge within the tested range of rainfall intensity (0.3–0.5 cm/min), and time ratio. This effect had occurred due to the increase in infiltration and resistance to the flow as observed by Mu et al.^50^. Also, there is an extremely weak relationship between rainfall intensity and peak discharge, with an R value of 0.09. This is following Lann et al.^51^ who reported that runoff generated by rainfall depends on the amount of rainfall on hilly terrain and would depend on flexible vegetation. When the input parameters are analyzed individually, the results of the correlation heatmap indicate the smaller impact of slope, time ratio, and rainfall intensity of the runoff, while flexible vegetation has a very important role in the hydrological response. Heatmap obtained results showed that flexible vegetation yields to reduction of peak discharge on hilly terrain by increasing infiltration rate and contributes to a sustainable flood mitigation framework. The negative relationship between the ratio between time and the concentration time (T/Tc) and the peak discharge (as seen in both flex and rigid vegetation conditions) also supports the results found by Jahanshahi and Booij^49^, who have also illustrated that the longer the time of concentration in a vegetated catchment, the later the peak discharge will be achieved. Likewise, Mu et al.^50^ described that vegetated slopes with very low gradients contribute to a decrease in acceleration on surface flows. The small relationship between slope and rainfall intensity in this work is explained by the small range of the experiment (0°–2° and 0.3–0.5 cm/min), which was also indicated in experimental works by Rehman et al.^43^. This supports the sensitivity of peak discharge to time in flat areas with vegetative cover.

In the case of rigid vegetation, a correlation heatmap of input and output parameters is shown in Fig. 3b. The result shows that there exists a negative correlation (with an R value of −0.45) of the time ratio and peak discharge against rigid vegetation conditions, resulting in less runoff generation on hilly terrain. Results demonstrate that even if the vegetation is rigid, it offers resistance to the flow, which is less than that for flexible vegetation conditions^52^. Moreover, the minimum influence of the terrain slope on the peak discharge is observed from an R value of 0.16. The flow disruption resulting from the rigid vegetation creates a reduction of the flow velocity^53^. However, the rainfall intensity has only a weak relationship with peak discharge (*R* = 0.15), indicating that the maximum hilly terrain is developed with a moderate influence of rainfall intensity on peak discharge from the hills. Finally, comparison showed that terrain slope has a greater influence on the peak discharge compared to rainfall intensity and time ratio in the conditions of rigid vegetation and according to the results found in the literature^54^. The result indicated that rigid vegetation gained a much smaller reduction in peak discharge compared to flexible vegetation, which results from the higher infiltration capacity of the flexible vegetation. In this study, a smaller variation of the slope (0°–2°) and rainfall intensity (0.3–0.5 cm/min) was utilized, which limited their influence on the precise correlation with peak discharge generated in a hilly terrain. This smaller variation in these parameters may impact the statistical sensitivity, influencing the correlation with peak discharge. Consequently, although the heatmap demonstrates a better correlation with the time ratio, the lower correlations when it comes to slope and rainfall intensity might not present the full effect that the latter two have in the real world when considered in more general circumstances. This drawback implies that future research should consider a larger scope of hydrological inputs.

### 10-fold cross validation

In this study, 10-fold cross validation was performed under rigid and flexible vegetation for random forest and support vector regression models, indicating different performance assessed through metrics including RMSE, MAE, Nash-Sutcliffe Efficiency (NSE), and Kling-Gupta Efficiency (KGE) as summarized in Tables 3 and 4. The 10-fold cross validation was performed the assess the performance and reliability of each model under rigid and flexible vegetation conditions. In this section, the result of 10-fold cross-validation for both models under rigid and flexible vegetation is explained separately. The result of 10-fold cross-validation in the case of the SVR model shows a notable variation in the values of performance metrics, demonstrating model sensitivity to hyperparameter tuning and the distribution of data series. The variation of the NSE, RMSE, and MAE values of the SVR model ranged between 0.011 and 0.313, 15.264 to 21.284, and 12.619 to 17.465, respectively (Table 3). This variation of performance metrics of the SVR model suggests considerable inconsistency with moderate predictive precision of the model. The KGE values of the SVR model across 10-fold cross-validation vary from 0.008 to 0.222, demonstrating significant instability of the model. The KGE value of the SVR model under fold 6 indicates the model’s capability of capturing complex hydrometeorological dynamics. However, in the case of flexible vegetation scenarios, the SVR model demonstrates slightly better performance with NSE, RMSE, and MAE values ranging between 0.044 and 0.350, 12.090 to 20.189, and 8.569 to 14.829. The KGE values of the SVR model in the case of flexible vegetation ranged between 0.044 and 0.282. This suggests that the SVR model is struggling with a generalization issue across various conditions of rainfall and terrain. Further, the variation of the performance metrics values may be due to the dependence of the SVR model on kernel functions and their sensitivity to outliers. This shows that the SVR model is not very significant while dealing with nonlinear hydrological phenomena.

In contrast, the random forest model has significant stability and performance across all 10 folds, demonstrating robustness of strong predictive capability while dealing with complex data series of interaction between vegetation and flow, as summarized in Table 4. The NSE and KGE values of the RF model in the case of rigid vegetation ranged from 0.898 to 0.968 and 0.846 to 0.962, respectively (Table 4). Further, other metrics such as RMSE and MAE values ranged from 3.261 to 7.380 and 2.291 to 5.210. The RF model has the highest values of NSE (0.968) and KGE (0.954) under rigid vegetation in the case of fold 10. The result demonstrates significant predictive accuracy of the RF model to capture a complex relationship between rainfall intensity, slope, and vegetation effects. The result of the RF model in the case of flexible vegetation shows a slight variation with an NSE value of 0.650 and an RMSE value of 9.481 across fold 8 of the k-fold cross-validation. Despite this, the RF model demonstrates excellent performance with an NSE value of 0.953 and a KGE value of 0.973 across fold 2, indicating the reliability of the model in predicting a complex hydrological phenomenon. The superior consistency that RF has over SVR is because RF is an ensemble method, and thus will likely decrease overfitting by averaging the multiple decision trees. The comparative analysis points out that RF is a more reliable model not only in rigid, but also in flexible vegetation, especially under hilly terrain where nonlinear interactions are predominant. Although SVR has sometimes shown good performance, its imprecision does not recommend it for actual use without elaborate tuning. The robustness of RF to even complicated circumstances also indicates that it is more suitable in the field of hydrological modeling, where it becomes essential to generalize.

**Table 3:** Summary of 10-fold cross-validation using a support vector regression (SVR) model for flexible and rigid vegetation.

Fold	SVR (RV)				SVR (FV)			
	RMSE	MAE	NSE	KGE	RMSE	MAE	NSE	KGE
1	21.284	17.165	0.233	0.195	16.114	11.858	0.264	0.245
2	15.264	13.642	0.277	0.222	12.090	10.197	0.219	0.151
3	21.012	16.068	0.051	0.072	20.189	14.367	0.124	0.114
4	17.492	13.185	0.124	0.206	13.547	10.055	0.054	0.122
5	19.792	17.465	0.011	0.145	16.805	14.233	0.161	0.044
6	17.088	12.940	0.056	0.008	19.631	14.829	0.044	0.120
7	20.135	16.416	0.244	0.185	16.059	11.428	0.244	0.232
8	19.252	16.494	0.263	0.197	13.322	11.037	0.309	0.254
9	15.969	12.619	0.313	0.214	12.217	8.569	0.350	0.282
10	17.617	15.063	0.252	0.213	18.047	12.944	0.254	0.194

**Table 4:** Summary of 10-fold cross-validation using a random forest (RF) model for rigid and flexible vegetation.

Fold	RF (RV)				RF (FV)			
	RMSE	MAE	NSE	KGE	RMSE	MAE	NSE	KGE
1	6.268	4.551	0.933	0.880	4.977	3.455	0.930	0.893
2	3.261	2.789	0.967	0.947	2.962	2.418	0.953	0.973
3	5.143	4.124	0.943	0.920	8.951	6.208	0.828	0.709
4	5.585	4.053	0.911	0.913	6.110	4.911	0.808	0.870
5	5.497	4.413	0.924	0.962	6.930	5.070	0.803	0.898
6	4.475	3.520	0.935	0.869	7.743	5.277	0.851	0.870
7	7.380	5.210	0.898	0.904	6.606	3.613	0.872	0.929
8	6.408	4.637	0.918	0.846	9.481	4.316	0.650	0.624
9	5.713	3.859	0.912	0.929	4.418	2.865	0.915	0.928
10	3.669	2.291	0.968	0.954	4.093	2.548	0.962	0.915

### Models performance

**Fig. 4:**
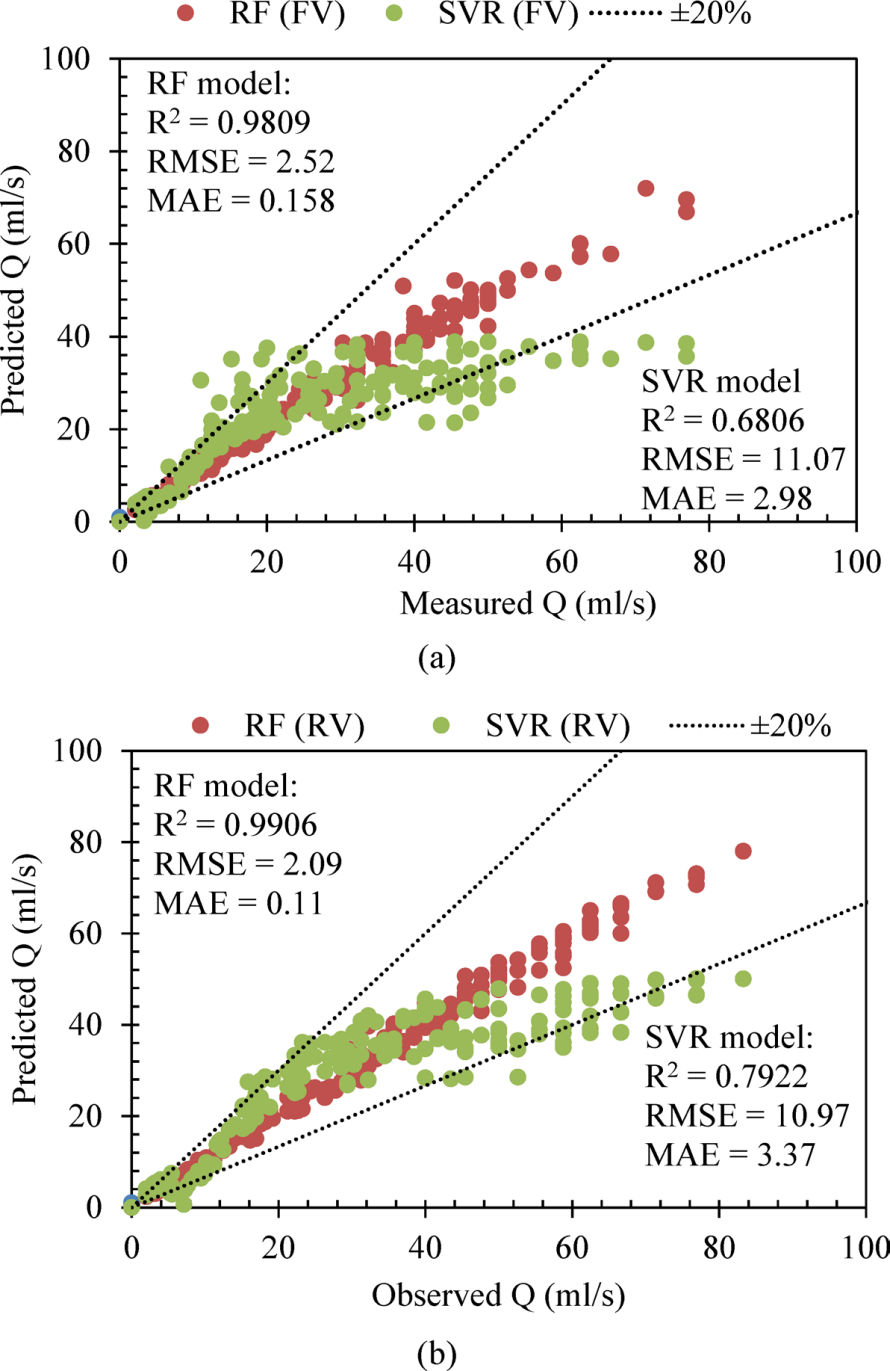
Predicted values of the peak discharge utilizing random forest and support vector regression models for flexible and rigid vegetation conditions (**a**) for flexible vegetation (FV) (**b**) for rigid vegetation (RV).

For predicting the peak discharge under diverse vegetation conditions including flexible (FV)and rigid vegetation (RV), performance of AI models were assessed by demonstrating values of the coefficient of determination (R^2^), root mean square error (RMSE), and mean absolute error (MAE) as shown in Fig. 4a, b. Figure 4 depicts the combined predicted runoff from all phases, including training (70%), testing (15%), and validation (15%). The purpose of this plot was to visualize the overall predictive power of the AI models across the data series utilized in the present study. Although the performance of models was evaluated during every stage to ensure the model’s generalizability and avoid the overfitting problem, the plot (Fig. 4) was created to display the overall performance of the models. In this section, results of model performance are explained in two different phases, that is, for flexible vegetation and rigid vegetation conditions. In the first phase, performance of the RF and SVR models was assessed for flexible vegetation conditions employed in a hilly terrain. The result demonstrates an R^2^ value of 0.9809 for the RF model while an R^2^ value of 0.6806 was achieved for the SVR model (Fig. 4a). This shows the superior performance of the RF model over the SVR model. This is due to the capability of the RF model to capture complex and nonlinear associations between input and output parameters of the hydrological phenomenon, and a similar result was reported in previous research^55^. This also shows that the RF model has the capability of developing relationships between various parameters such as peak discharge with rainfall intensity, Time ratio, and terrain slope. In this study, the Nash-Sutcliffe Efficiency (NSE) and Kling-Gupta Efficiency (KGE) have been assessed for RF and SVR models in the case of rigid and flexible, for a better understanding of the model reliability and variability. The results demonstrate the NSE values of 0.9804 and 0.9902 for the RF model in the case of flexible and rigid vegetation, while KGE values for both vegetation conditions by using the SVR model assessed were 0.9666 and 0.9751, respectively. Furthermore, the NSE and KGE values of the SVR model for flexible vegetation were 0.4985 and 0.5051, and in the case of the rigid vegetation scenario, the values (NSE and KGE) were 0.6849 and 0.6066, respectively. The findings from the NSE and KGE values show the superior performance of the RF model over SVR, because of the ensemble nature of the RF model in handling complex relationships between the data series and the dependence of the SVR model on kernel selection and regularization.

Performance superiority of the RF model under FV conditions is owing to its ensemble learning design, which produces average results from multiple decision trees to minimize overfitting and enhance accuracy as reported by^56^. The SVR model finds it difficult to reproduce complex patterns in the data since variations in flow patterns resulting from vegetation interactions decrease its ability to generalize information. The wide praise in hydrological modeling and robustness of RF is supported by literature^57^ specifically in vegetated terrains, because flexible stems create temporal runoff flow redistribution through dynamic resistance. Flexible vegetation reduces peak discharge by creating more rough surfaces and increasing water absorption^52^ whereas the modeling ability of RF is effective. The RF model shows better performance due to its structure in assessing temporal variations of hydrodynamic resistance, since Flexible vegetation adapts by bending during fluid interactions. The R^2^ value from SVR models signifies substantial challenges to capture flow dynamics combined with adaptive vegetative resistance because its kernel limitations and hyperparameter selection might not be optimized for these dynamic conditions.

The performance metrics show improved outcomes for both models when examining rigid vegetation (RV) even though the results exceeded flexible vegetation scenarios with the SVR model achieving R^2^ = 0.7922 compared to its initial value of R^2^ = 0.6806 (Fig. 4b). The RF model presents superior prediction capabilities through its exceptional R^2^ value of 0.9906. A random forest (RF) model had higher R^2^ values of 0.9809 and 0.9906 with flexible and rigid vegetations, respectively, as compared to the SVR model, with R^2^ values of 0.6806 and 0.7922. This was shown to be in line with Bargam et al.^37^ and Granata et al.^36^, who demonstrated that the RF was more effective in dealing with nonlinearity in the hydrological data compared to kernel-based SVR when complex vegetation-induced flow occurred. Improved performance regarding rigid vegetation can probably be attributed to more stable hydraulic resistance, which was found similarly by Meddage et al.^38^ in their study of vegetated open-channel flows. The regular hydraulic resistance exerted by rigid vegetation appears to make runoff characteristics more foreseeable when compared to flexible vegetation. Rigid vegetative stems that maintain straight positions when flow passes through produce dependable surface pathways and dissipation systems for water energy as reported by^58^. Both the simplicity of the SVR model and its ability to detect patterns become possible because rigid vegetation structure decreases flow resistance fluctuations. Literature supports rigid vegetation modeling through steady drag effects because AI models can properly represent the data when the defined input features, such as terrain slope, rainfall intensity, and time ratio, exist^38^. RF’s ensemble design enables precise hydrodynamic modeling of multiple parameters through its ability to identify relationships between features, making it achieve reliable generalization under data uncertainty. Despite robustness, the random forest model can be susceptible to overfitting issues, particularly when applied to small data series. However, in this study, the overfitting issue was encountered by adapting various strategies; (1) to ensure unbiased evaluation of the model performance, the data series was split into three different phases, including training (70%), testing (15%), and validation (15%) suggested by Xu and Goodacre^44^, (2) maximum depth and number of trees were optimized for performing hyperparameter tunning, which prevent fitting noise of the excessively complex tree in a data series, and (3) the ensemble nature of RF inherently reduces overfitting by averaging multiple decision trees trained on different bootstrapped samples. Generalization of the model was also supported by the even distribution of errors, showing the stability and minimum variance of the results. All these measures increased the model’s reliability level and reduced the risk of overfitting. Rigid structures provide the system with more reliable hydraulic responses because they help reduce output feature (peak discharge) variability, which leads to improved SVR performance under RV modeling. The performance evaluation demonstrates that vegetation type stands as an essential controlling factor that affects the flow dynamics of the model. Model selection with artificial intelligence requires examination of both architectural structures alongside land surface, vegetative, and hydraulic behavior dynamics for precise peak discharge estimation under different land cover conditions during storms.

### Error distribution

**Fig. 5:**
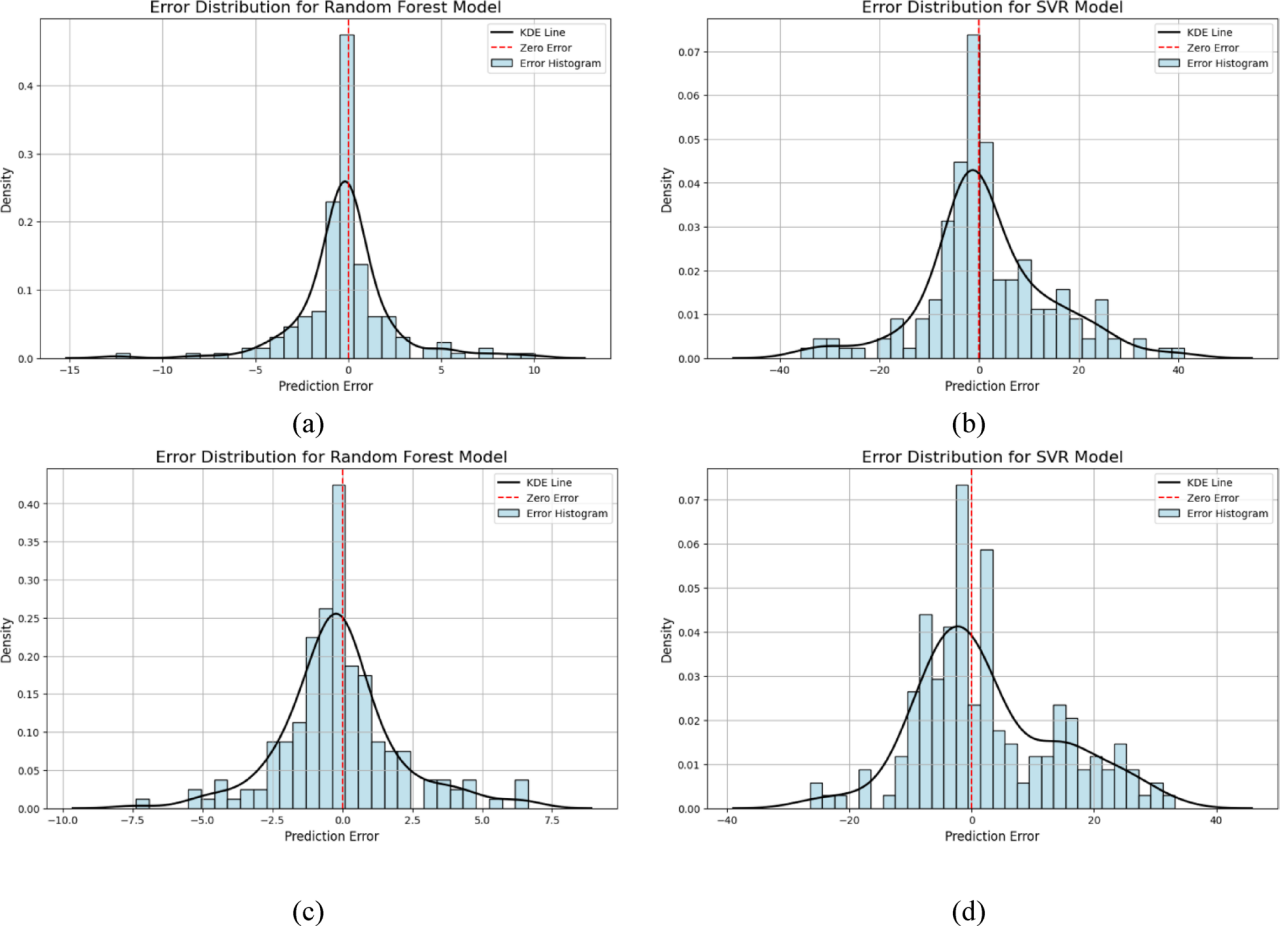
Error distribution plot for random forest (RF) and support vector regression (SVR) model under flexible (FV) and rigid vegetation (RV) conditions (**a**) RF model for FV (**b**) SVR for FV (**c**) RF for RV (**d**) SVR for RV.

The performance of the RF and SVR models was assessed using the Kernel Density Estimation (KDE) line and histogram through error distribution analysis in the case of flexible and rigid vegetation conditions, as shown in Fig. 5a–d. In the case of flexible vegetation, RF and SVR provide different performance characteristics. The error distribution of the RF model shows an error concentration between − 15 to + 10, as shown in Fig. 5a. The higher accuracy of the RF model in the prediction of the peak discharge can be seen from the symmetry around zero error, indicating minimum bias and a greater value of density near zero. The result of the RF model demonstrated superior performance by developing a relationship between nonlinear and complex hydrological responses as well as reducing the overfitting issue of the model, as reported by Akbar et al.^59^.

The SVR model for flexible vegetation shows a wider error distribution from − 40 to + 40 while having a reduced peak near zero error, based on Fig. 5b. The SVR model generated this outcome because its complex nature applied to non-linear data linking while being unaffected by kernel functions, as well as sensitive to tuning parameters. The SVR model performs less consistently while modeling flexible vegetation behavior because of its complicated relationship with runoff systems, according to Kuter^60^. The RF model achieved superior peak discharge prediction performance because it demonstrated better forecasting abilities in flexible vegetation conditions. Further adjustments to the SVR model will lead to similar performance outcomes as the RF model showcases at this time.

The evaluation of performance for the RF and SVR models under rigid vegetation conditions included their error distribution presented in Fig. 5c, d. The error distribution for RF models under flexible vegetation conditions spread from − 10 to 7.5, while the dense peak is located at zero (0.40), indicating minimum bias alongside strong precision and accuracy of the RF model. The research results show that the RF model provides a superior understanding of hydrological process responses to rigid vegetation because it can handle non-linear data patterns aligns with the finding of Kumar et al.^61^. The SVR model generates predictions with variable density, which produces a wide range of error predictions. While the SVR model demonstrates limited success in reproducing hilly geomorphology peak discharge patterns it exhibits effective capabilities for modeling runoff behavior according to the vegetation rigidity level (Granata et al.^36^ reported this finding in their rainfall-runoff modeling incident with SVR). The error distribution plots also support the robustness of the RF model, where the major concentration of errors occurred within the limits of about ± 10 during flexible conditions and ± 7.5 during rigid conditions. This symmetry complies with the observations made by Akbar et al.^59^, who found negligible errors in the residual bias of RF forecasts of flood-related indicators. The broader error spread in SVR (± 40) indicates its insensitivity to dynamic vegetative resistance, which is an issue that Kuter (2021) has found in the terrain modeling of the SVR. Flexible vegetation comparison using the RF method generates results similar to those of rigid vegetation; however SVR model demonstrates inferior performance and larger error ranges in rigid vegetation conditions. This shows that rigid vegetation offered greater resistance to the flow resulting from complex and nonlinear patterns of data series that cause the SVR model’s generalization capability. The result offered a superior performance of the RF model in reducing the effect of hydrological processes in a vegetated environment on hilly terrain.

### SHAP analysis

**Fig. 6:**
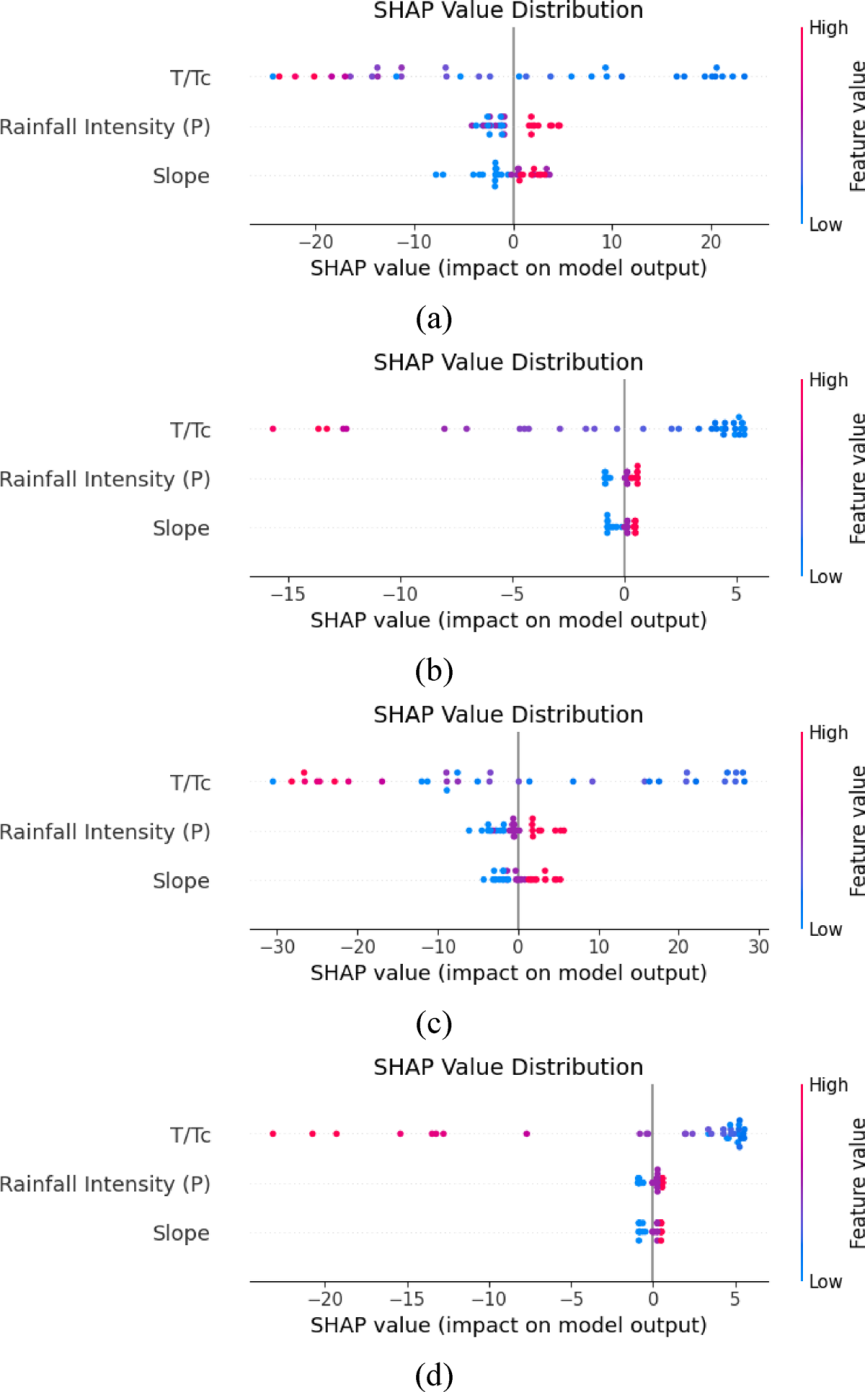
SHAP analysis for random forest and support vector regression model under various hydrological and vegetation conditions (**a**) RF model for flexible vegetation (**b**) SVR model for flexible vegetation (**c**) RF model for rigid vegetation (**d**) SVR model for rigid vegetation.

SHAP analysis provides the influence of the input parameters, such as time ratio, rainfall intensity, and terrain slope, on the prediction of output parameters (peak discharge) by the RF and SVR under flexible and rigid vegetation conditions. Figure 6a-d depicts the result of SHAP analysis in the case of flexible and rigid vegetation conditions using RF and SVR models. In the case of flexible vegetation, it was observed that the time ratio has a significant influence on the prediction of peak discharge by utilizing the RF model, indicating high variability in SHAP values (−20 to 20) as depicted in Figure (6a). Further, the case of the RF model shows a higher impact of rainfall intensity resulting in greater runoff through flexible vegetation conditions, and a similar trend was observed by Rehman et al.^43^. They observed that by increasing the rainfall intensity, runoff generated from the hilly terrain increased while keeping terrain slope and time ratio constant. Following the rainfall intensity, it was observed that the terrain slope has a minimum influence on the generation of peak discharge, as shown in Fig. 6a. However, in the case of the SVR model for flexible vegetation, it was observed that the time ratio shows less sensitivity (SHAP values ranged between − 15 and 5) to the prediction of the peak discharge compared to the RF model (Fig. 6b). Furthermore, the SVR model shows a negligible impact of terrain slope on the prediction of the peak discharge. Also, the SVR model shows a slightly higher impact of rainfall intensity on the prediction of peak discharge compared to terrain slope and a lower than time ratio. This result was obtained due to the poor kernel-driven function capability of the SVR model to understand the complex and nonlinear interaction between various variables. Upon comparing the results of the RF and SVR models, it was observed that the RF model shows slightly better performance than the SVR model. Further, it was observed that time ratio acts as a primary factor influencing the prediction of the peak discharge, while rainfall intensity and terrain slope play secondary roles in the case of flexible vegetation conditions. The result also recommends the significance of the RF model while modeling vegetation-impacted hydrological processes. SHAP values also suggested that time ratio was the strongest characteristic under both vegetation types, with the range of values at ± 28 (FV) and − 30 to + 28 (RV), which confirms the conclusion of Wang et al.^39^, according to which time ratio was indicated as one of the controlling forces in runoff delay in AI based flood models. The increased SHAP value plays under flexible vegetation indicates a higher interaction with temporal hydrodynamics, indicating that the argument by Liu et al.^9^ questioning whether rigid or flexible vegetation is better in accomplishing temporal buffering mechanisms and thus in altering peak flow is correct, since flexible vegetation alters peak flow through its temporal buffering mechanisms more effectively in comparison with rigid structures.

In the case of rigid vegetation conditions, the SHAP analysis by using RF model, indicates how altering vegetation type influenced input parameters on the prediction of the peak discharge, as shown in Fig. 6c. Similar to the flexible vegetation conditions, it was observed that the time ratio is the most influential factor affecting the prediction of the peak discharge with SHAP values (−30 to 30). However, the SHAP value range in the case of rigid vegetation increased from − 30 to 30. This means that rigid vegetation increases runoff time due to the resistance offered by the trunk part of the rigid vegetation, as previously reported by Murtaza et al.^62^. Furthermore, terrain slope has a greater contribution to the direct runoff generated in the case of rigid vegetation compared to flexible vegetation scenario. Also, rainfall intensity has moderate impact on the peak discharge compared to the time ratio and is greater than the slope. This happened because, in the case of rigid vegetation, the trunk part only resists the flow velocity and has limited or no infiltration capability. Moreover, for the SVR model in the case of rigid vegetation conditions, SHAP analysis shows a lower impact of the time ratio (SHAP value ranged from − 20 to 5) compared to flexible vegetation conditions (Fig. 6d). The terrain slope and rainfall intensity has almost zero influence. This result demonstrates that the SVR model struggles with complex and nonlinear patterns of the data series to enhance resilience on the kernel function, which mostly oversimplifies such types of relationships. Upon comparing the results of flexible and rigid vegetation conditions, it was observed that flexible vegetation performed better than rigid vegetation conditions because of their resistance to the flow and higher infiltration capability of the flexible vegetation. SVR’s effectiveness weakens more under restricted circumstances, but RF retains excellent understanding across different kinds of vegetation, confirming RF’s dominance in managing vegetation-mediated hydrology. With flexible vegetation displaying greater plant-dominated constraints and rigid vegetation displaying traditional runoff dynamics, these discrepancies highlight how vegetation flexibility significantly alters parameter relevance in hydrological modeling. Previous studies reported that rigid vegetation types like trees and shrubs with deeper root systems improved water absorption by infiltration and soil permeability^12,51^. However, in the current study, the root system of the rigid and flexible was not considered; instead surface level of the vegetation (flexible and rigid) was adopted under a controlled laboratory setting. For this purpose, Rehman et al.^43^ adopted rigid vegetation as wooden branches and flexible vegetation as grass mats. The objective of these proxies was to ensure that they modeled surface resistance and overland flow dynamics, thus showing that the resultant high performances on peak discharge reduction by flexible vegetation cover are attributed to the influence of an increase in surface roughness and detention aspects as compared to rigid vegetation. A quantitative comparison of SHAP values shows a clear difference between the feature contribution of RF and SVR models in the case of both vegetations. The RF model presents the range of the SHAP values of T/Tc to be broader (−22 to + 22) with flexible vegetation than with the SVR and a narrower range (at −15 to + 5), confirming less sensitivity towards the variable. In the case of rigid vegetation, again, RF has a large calculated SHAP range, and T/Tc (−30 to + 30) as opposed to SVR having a range of approximately − 20 to + 6. In general, SHAP variability was higher in the RF model than in SVR in all of the cases.

### Cumulative accuracy profile (CAP) curve

**Fig. 7:**
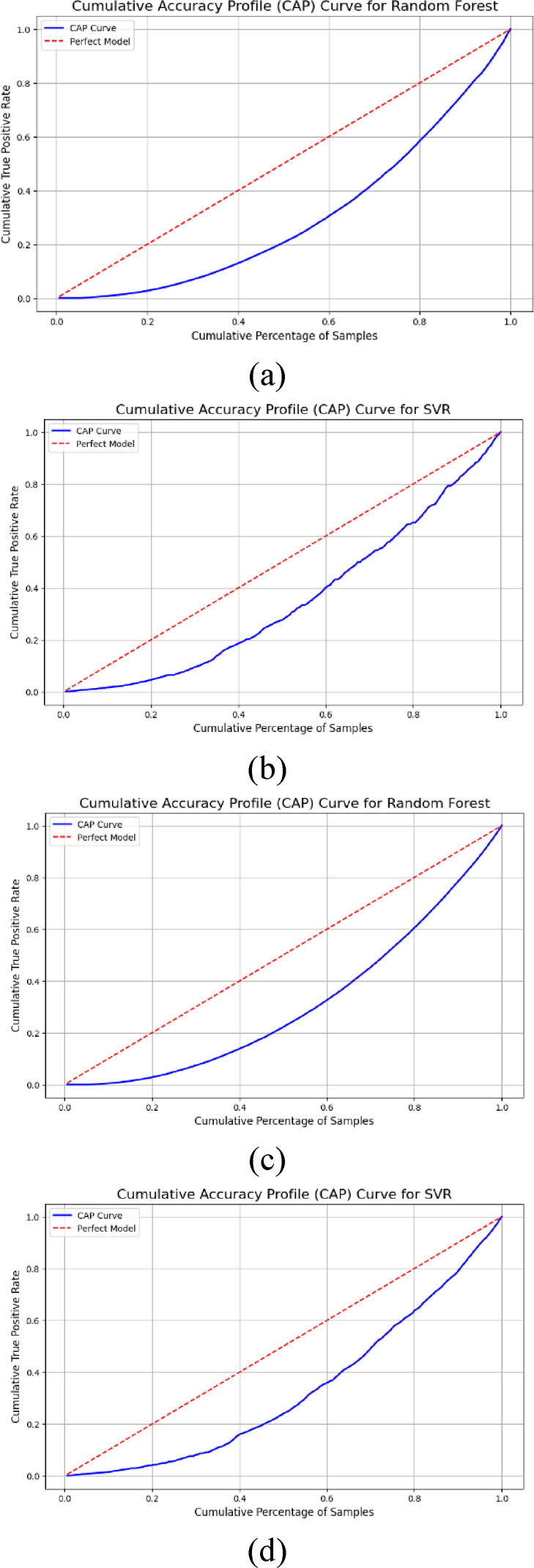
Cumulative accuracy profile for AI models utilizing flexible (FV) and rigid (RV) vegetation (**a**) RF for FV (**b**) SVR for FV (**c**) RF for RV (**d**) SVR for RV.

The predictive capability of the AI models, such as RF and SVR, was observed utilizing the cumulative accuracy profile (CAP) curve for flexible and rigid vegetation conditions as shown in Fig. 7a–d. The result of the CAP curve demonstrated the superior performance of the RF model over the SVR model due to the steeper rise and larger area covered between the baseline and the model curve. In contrast, the CAP curve captures the weaker performance of the SVR model in predicting peak discharge in the case of flexible vegetation (Fig. 7a, b). The RF model delivers higher cumulative true positive detection across a reduced subset of data samples, upholding its strength for hydrological model analysis under vegetation factors. RF demonstrates superior performance compared to SVR in this situation, mainly because its ensemble structure and its advanced handling of feature interactions are better adapted to hilly terrain.

The SVR and RF prediction models produce their peak discharge prediction results through CAP curves for rigid vegetation conditions on hilly terrain using time ratio, rainfall intensity, and terrain slope input parameters as shown in Fig. 7c, d. The CAP curve from the RF model exhibits a rapid performance boost in cumulative true positive rates, which almost reaches its perfect model line due to its high predictive accuracy. RF demonstrates strong capabilities to uncover non-smooth dependencies between peak discharge and its independent variables through its ensemble learning methodology. The CAP curve of the SVR model shows a gradual ascent, which indicates worse predictive efficiency when compared to the RF model. The difficulty of tuning SVR parameters and using kernel functions^63^ possibly fails to capture terrain-specific dynamics within rigid vegetation. RF produces better predictions because it demonstrates favorable robustness for modeling hydrological processes in challenging environments, therefore making it a more dependable option for peak discharge assessment in hilly regions with rigid vegetation. Hydrological modeling needs appropriate machine learning techniques for operational success because it demands accuracy and reliability in flood risk assessment^64^.

### Monte Carlo simulation (MCS) analysis

**Fig. 8:**
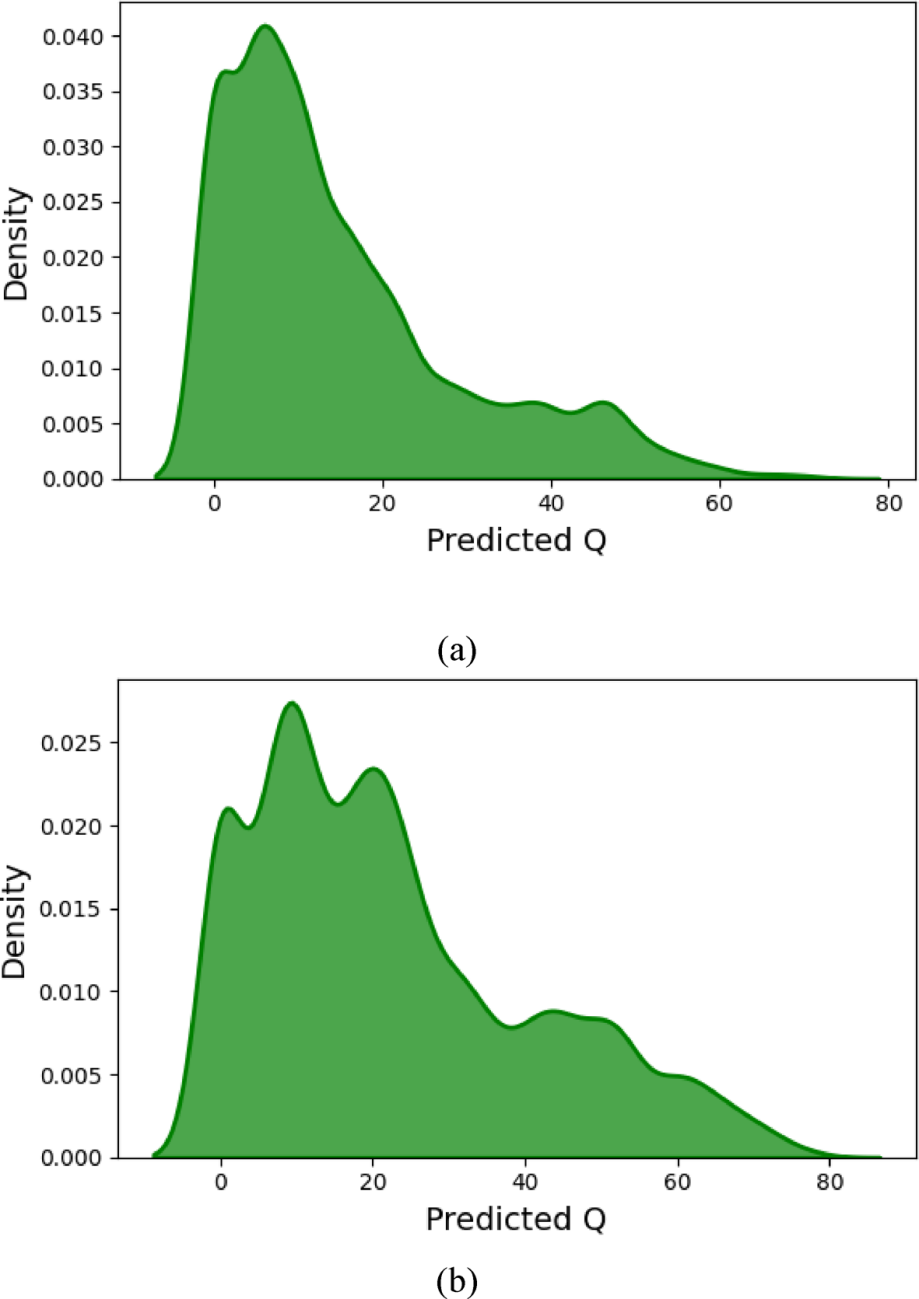
Monte Carlo Simulation of the peak discharge for various types of vegetation (**a**) for flexible vegetation (**b**) rigid vegetation.

The Monte Carlo Simulation (MCS) predicted the chance of occurrence of various outcomes by repeating samples randomly sampling to analyze uncertainty and optimize decisions. In the current research, MCS was performed to understand the probability of predicted peak discharge density in the case of flexible and rigid vegetation conditions (Fig. 8a, b). First, the MCS analysis is explained for flexible vegetation scenarios utilizing input parameters such as rainfall intensity, terrain slope, and time ratio. The result for flexible vegetation demonstrated the distribution of the peak discharge with a unimodal pattern and moderately spread over the Kernel Density Estimate (KDE) plot (Fig. 8a). The highest concentration bandwidth surrounding the central area demonstrates that the system will achieve stabilization when operated in this specific range of conditions. The distribution includes noticeable probabilities of extreme discharge events along its tails that may develop from multiple factors, including steep slopes combined with high rainfall intensity and critical time ratios. Hydrological modeling benefits from probabilistic approaches since deterministic approaches fail to identify these rare but extreme scenarios^40^. The research demonstrates that hilly regions with flexible vegetation require strong flood mitigation responses, given their discharge variability impacts on erosion processes and sediment movement.

The rigid vegetation model on hilly terrain shows through Monte Carlo simulation that its predicted peak discharge (Q) extends higher while maintaining a wider and gentler curve than flexible vegetation results. Q shows increased variability along with longer tails because flexible vegetation has worse flow attenuation capabilities when peak density falls to 0.025, while it stands at 0.040. In MCS results, both the presence of a unimodal, peaked discharge pattern with flexible vegetation and a wider, right-skewed discharge pattern with rigid vegetation corresponded with those reported by Turkel et al.^40^, who concluded that rigid, low-porous surfaces exhibited more variable runoff characteristics during very strong precipitation. This is in support of the view that a flexible vegetation system offers more predictive discharge behavior under different hydrological circumstances. Rigid vegetation, including woody shrubs and engineered barriers unlike inflexible vegetation produces water deflection along with turbulent areas that result in accelerated water runoff, particularly in conditions with high rainfall intensity combined with steep slopes. The right-skewed distribution reflects extreme discharge events that occur because of this behavior. Hilly areas risk raised peak discharge variability when employing rigid vegetation to control erosion, since such measures seem to increase these risks, which requires implementing additional solutions such as terracing and retention basins to manage floods^65^. A widespread uncertainty demonstrates why probabilistic modeling should be used for hydrological risk assessment in these systems.

### Permutation feature importance

**Fig. 9:**
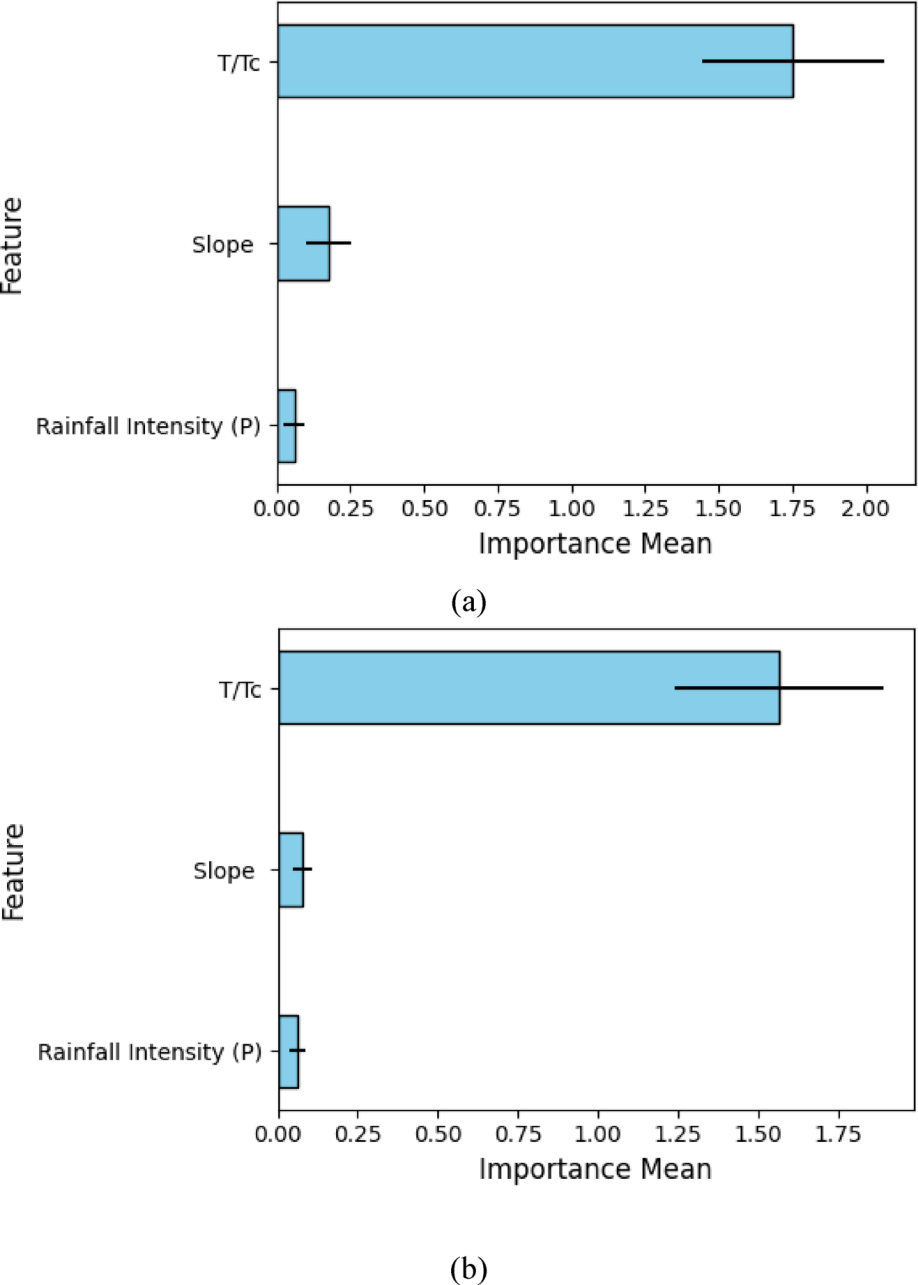
Permutation feature importance for the random forest model under flexible and rigid vegetation conditions (**a**) for flexible vegetation (**b**) for rigid vegetation.

The permutation feature importance graph was plotted for the best model (RF model) in the case of flexible and rigid vegetation conditions on hilly terrain (Fig. 9a, b). Although the result of the correlation heatmap presented in Fig. 3 and permutation feature importance (Fig. 9) provides insight into the relationship between input variables and peak discharge, they demonstrate different analytical perspectives. The correlation heatmap in Fig. 3 shows statistical relationships between variables where associations are being studied as linear, pairwise, irrespective of a modeling framework. Conversely, the permutation feature importance of Fig. 9 is dependent on the model and independent of an interpretation of the influence of each feature on the predictive performance of the trained model. It is done by quantifying the increment in model error of any variable arbitrarily changed, so capturing non-linear interaction. The approach is an addition to the correlation analysis that provides a more solid insight into the feature relevance within the AI framework, which supports the importance of including the approach to increase the interpretability and transparency of the model. The plot shows the importance of the input parameters in predicting the peak discharge in hilly terrain in terms of mean and standard deviation. The value of the mean is represented in chart form, while that of the standard deviation is in the form of an error bar as shown in Fig. 9a, b. The findings demonstrated the critical impact of time ratio (T/Tc), followed by terrain slope and rainfall intensity under rigid vegetation conditions (Fig. 9a). However, the trend of parameter influence observed in the case of flexible vegetation conditions was somewhat different. In the case of flexible vegetation, the critical parameter observed was terrain slope, followed by time ratio (T/Tc) and rainfall intensity. The plots of permutation importance prove the overall supremacy of T/Tc and slope in flexible systems, whereas the rigid systems are more susceptible to T/Tc. Such trends are in line with the principles of the vegetation-soil interaction presented in Lann et al.^51^ and Croke et al.^65^, in which slope-reactive vegetative cover types, such as grasses, increase infiltration and postpone the start of runoff better. The result of the current research recommended that vegetation has a significant role in altering hydrological phenomenon dynamics under diverse conditions. The importance measures of rigid vegetation show maximum values at T/Tc equal to 1.75, Slope at 1.25, and Rainfall Intensity at 0.75. The conditions for flexible vegetation result in Slope scoring 1.50, while T/Tc maintains a value of 1.00 and Rainfall Intensity stays at 0.50. The rigid vegetation models show greater emphasis on temporal ratios than flexible vegetation models do on topographic elements. Rainfall Intensity displays consistent low priority as a peak discharge predictor in both study plots. Rigid vegetation has significant dependence on T/Tc in the plot, which indicates its sensitivity to timing variables because of its unyielding structure that affects water velocities. Flexible vegetation has a relationship with Slope that demonstrates its capability to react to slope modifications while reducing temporal impacts. Hilly areas should use flexible vegetation because Slope measurement demonstrates its ability to adapt to different terrain conditions more efficiently than rigid vegetation that reflects strict temporal limitations.

### Partial dependence plots (PDP)

**Fig. 10:**
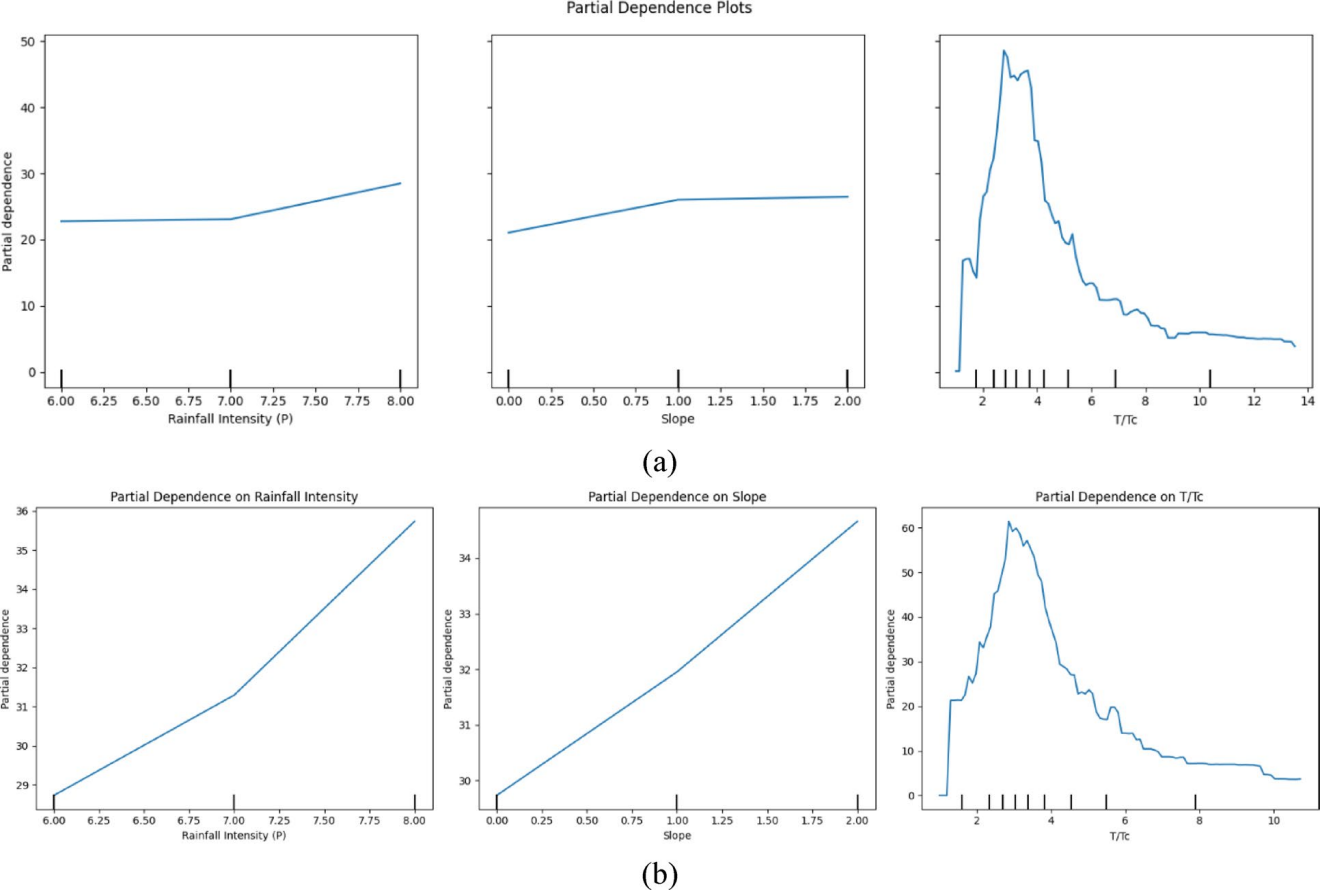
Partial Dependence Plots (PDP) for flexible and rigid vegetation conditions (**a**) for flexible vegetation (**b**) for rigid vegetation.

The PDP plots demonstrate that peak discharge rises directly with slope gradient in flexible vegetation systems, yet rainfall intensity (P) follows an accelerated non-linear pattern (Fig. 10a-b). The discharge patterns from rigid vegetation demonstrate non-linear increases when connected to rainfall intensity and slope levels, even when time ratios show random effects on behavioral discharge changes (Fig. 10b). The relationship between slope gradients and flexible vegetation shows consistent patterns, yet rigid vegetation shows the highest sensitivity to extreme rainfall and slope combinations. Peak discharge intensity in flexible vegetation increases steadily until 8.00 before rainfall intensity hits its maximum threshold, regardless of slope variation between 0.00 and 2.00. The discharge in rigid vegetation produces peaks that emerge when rainfall intensity exceeds 1.50, while the slope height stays above 1.25. The rigid vegetation pattern creates T/Tc curves with small yet noticeable peaks and dips because multiple complex temporal influences control its behavior rather than flexible vegetation. The smooth water discharge management of flexible vegetation surpasses rigid vegetation due to how its flexible reactions respond to high rainfall and slope gradients. Flexible vegetation performs better than rigid vegetation when it comes to flood control on hilly terrains since its slope-based reactions reduce floods more effectively than rigid vegetation does during extreme conditions (Fig. 10a).

### Research limitations and future directions

This research contains various limitations that help explore AI models and their effect on flood control between rigid and flexible vegetation on slopes. The laboratory experiments performed for research purposes did not successfully replicate real-world environment conditions due to their failure at replicating diverse plant states and soil heterogeneity and intense climate characteristics^66^. The gathered measurements provide detailed information, but the study restricts its result applicability outside conditions of rainfalls between 0.3 and 0.5 cm/min with slope angles between 0° to 2°. Peak discharge functioned as the primary focus variable in the analysis, while the study failed to monitor important output metrics such as sediment transport and groundwater recharge, and permanent vegetation destruction. The RF model displays excellent accuracy but requires considerable processing power and extensive training datasets, therefore posing difficulties to areas lacking technological resources^67^. Actual NBS implementation demands consideration of essential socio-economic factors such as implementation expenditure along with community preferences, even though the study neglected these vital features.

Further, future investigations should be carried out to fill the gap in the current research by the utilization of multi-scale approaches, integrating laboratory-scale experiments with field data replication under diverse climates and topographic conditions. Also, future research should expand the values of the terrain slope, rainfall intensities, and time ratio would improve the practicability of the models^68^. The predictive efficiency of the AI models can be improved by the utilization of additional parameters like soil type, vegetation density, thickness, and land-use changes. Integration of AI with hydrodynamic models should be explored for the utilization of a hybrid modeling framework to understand the deeper interplay between vegetation types and peak discharge. Furthermore, hybrid AI models including SVR with particle swarm optimization (PSO), adaptive neuro-fuzzy inference system (ANFIS) with PSO, artificial neural network (ANN) with PSO, and decision tree with the neural network should be utilized for a deeper understanding of the AI models predictive capability. Long-lasting observation systems of NBS implementation need to be maintained because they are instrumental in evaluating their resistance to climate change effects^69^. Research of socio-economic factors should explore the cost-benefit ratios and public reception and policy restrictions that will enable the expansion and accessibility of solutions. The deployment of NBS systems will receive better stakeholder collaboration and trust because of advanced explainable AI (XAI) interpretability tools, which form a bridge between technical outputs and decision-making. Future work should tackle these weak points because their improved AI-driven NBS strategies will lead to better flood management systems globally.

## Conclusion

In this paper, artificial intelligence techniques were utilized to assess the effectiveness of the NBS, particularly flexible and rigid vegetation conditions on hilly terrain for mitigating flood risk. Artificial intelligence models such as random forest and support vector regression were employed to understand the response of NBS against peak discharge generated from hilly terrain under diverse hydrological conditions such as terrain slope, rainfall intensity, and time ratio. A significant reduction in the peak discharge was reported in the case of flexible vegetation due to higher infiltration capacity and flow resistance (trunk and crown part). Upon comparison of the AI models, it was found that RF has greater capability in handling complex relationships between input and output parameters, providing more accurate predictions of the peak discharge. The conclusion of the current investigation is as follows:

- The findings demonstrated that the utilization of flexible vegetation like grass mats significantly causes a reduction of peak discharge by slowing surface runoff, making it a more successful type of NBS in flood management in hilly regions. The flexible vegetation not only has resilience against varying terrain slopes and rainfall intensities but also shows greater effectiveness in biodiversity support and soil stabilization. The findings also demonstrated that a region prone to intense and heavy rainfall should utilize flexible vegetation due to its widespread adaptation capability against flash and rural flood resilience plans compared to rigid vegetation.
- The findings of the current research concluded the outperformance of the RF model over SVR in evaluating the response of NBS against varying hydrological frameworks, indicating the reliability of the RF model for flood risk modeling. The complex interaction between data series was captured significantly by the RF compared to the SVR model, reflecting the higher R^2^ value and lower RSME value. Therefore, the findings recommended that such an AI-driven approach should be utilized in early warning frameworks and flash planning systems for capturing real-world flood forecasting scenarios and optimization of NBS utilization.
- Nature-based solutions through flexible vegetation become essential for adapting to more severe rainfall patterns and increased flood prospects that result from climate change. Nature-based solutions that reduce extreme weather damage and recover ecosystems support the global sustainability targets, including the Sendai Framework for Disaster Risk Reduction of the United Nations. These solutions decrease dependence on fixed infrastructure to create lasting resilience, which also solves flash heat island issues and targets water shortages. Climate action plans should give priority to such systems for communities to develop better protection against upcoming hydrological uncertainties, which will reduce both environmental losses and economic impacts of flooding.

Future research on NBS should utilize an AI-driven approach for varying climatic and geographical conditions to validate the scalability of the current research. Furthermore, various additional input parameters such as land-use patterns, soil composition, and vegetation density should be included to improve the predictive capability of the AI-driven models. Flood simulation accuracy may be improved by combining AI with hydrodynamics modeling.

## Supplementary Information

Below is the link to the electronic supplementary material.

Supplementary Material 1

## Data Availability

The data is available from the corresponding author upon request.
