# Supplementary material for: Artificial intelligence evaluation of nature based flood resilience in hilly terrain
Source: Sci Rep. 2025 Oct 10;15:35492. doi: 10.1038/s41598-025-19629-9 (PMC12514235; doi:10.1038/s41598-025-19629-9)
Supplement: Supplementary file 1 — Supplementary Material 1 [file 41598_2025_19629_MOESM1_ESM.docx]

Supplementary materials

Correlation Heatmap Code:

# Install openpyxl to handle Excel files

!pip install openpyxl

# Import required libraries

import pandas as pd

import seaborn as sns

import matplotlib.pyplot as plt

from google.colab import files

# Upload the Excel file

uploaded = files.upload()

# Read the Excel file

file_name = next(iter(uploaded))

df = pd.read_excel(file_name, engine='openpyxl')

# Display the original column names

print("Original column names:")

print(df.columns.tolist())

# Clean column names: strip whitespace and convert to lowercase

df.columns = df.columns.str.strip().str.lower()

# Display the cleaned column names

print("\nCleaned column names:")

print(df.columns.tolist())

# Rename columns to standardized names

df.rename(columns={

    'rainfall intensity (p)': 'P',

    'slope   (%)': 'slope',

    't/tc': 'T_Tc',

    'q': 'Q'

}, inplace=True)

# Display the renamed column names

print("\nRenamed column names:")

print(df.columns.tolist())

# Select relevant columns

columns_of_interest = ['Q', 'T_Tc', 'slope', 'P']

df_selected = df[columns_of_interest]

# Compute the correlation matrix

corr_matrix = df_selected.corr()

# Set font to Times New Roman

plt.rcParams['font.family'] = 'Times New Roman'

# Create the heatmap

plt.figure(figsize=(8, 6))

sns.heatmap(corr_matrix, annot=True, cmap='coolwarm', fmt=".2f", linewidths=0.5)

plt.title('Correlation Heatmap for Peak Discharge and Independent Variables', fontsize=14)

plt.tight_layout()

# Save the heatmap as an image file

image_filename = "correlation_heatmap.png"

plt.savefig(image_filename, dpi=300)

plt.show()

# Provide the download link for the image

files.download(image_filename)

Partial Dependence Plots (PDP) Code

# %% [markdown]

"""

# Partial Dependence Plots (PDPs) Analysis

This notebook analyzes the relationship between rainfall intensity (P), slope, T/Tc (independent variables) and Q (dependent variable) using Partial Dependence Plots.

"""

# %%

# Install required packages

!pip install pandas numpy matplotlib scikit-learn plotly

# %%

import pandas as pd

import numpy as np

import matplotlib.pyplot as plt

from sklearn.ensemble import RandomForestRegressor

from sklearn.inspection import PartialDependenceDisplay

from sklearn.model_selection import train_test_split

from sklearn.metrics import mean_squared_error

# %%

# Load the data

# Since we're in Colab, we'll upload the file manually

from google.colab import files

uploaded = files.upload()

# %%

# Read the Excel file

file_name = list(uploaded.keys())[0]

df = pd.read_excel(file_name)

# Clean column names by stripping whitespace

df.columns = df.columns.str.strip()

# Display first few rows

print("Data Preview:")

print(df.head())

# %%

# Data preprocessing

# Check for missing values

print("\nMissing values:")

print(df.isnull().sum())

# Check data types

print("\nData types:")

print(df.dtypes)

# %%

# Prepare data for modeling

# Use the exact column names as they appear in your data

X = df[['Rainfall Intensity (P)', 'Slope', 'T/Tc']]  # Updated 'Slope (%)' to 'Slope'

y = df['Q']

# Split data into train and test sets

X_train, X_test, y_train, y_test = train_test_split(X, y, test_size=0.2, random_state=42)

# %%

# Train a Random Forest model

print("\nTraining Random Forest model...")

model = RandomForestRegressor(n_estimators=100, random_state=42)

model.fit(X_train, y_train)

# Evaluate model

train_pred = model.predict(X_train)

test_pred = model.predict(X_test)

print(f"Train RMSE: {np.sqrt(mean_squared_error(y_train, train_pred)):.2f}")

print(f"Test RMSE: {np.sqrt(mean_squared_error(y_test, test_pred)):.2f}")

# %%

# Create Partial Dependence Plots

print("\nCreating Partial Dependence Plots...")

features = [0, 1, 2]  # Indexes for 'Rainfall Intensity (P)', 'Slope', 'T/Tc'

# Standard PDP plots

fig, ax = plt.subplots(figsize=(15, 5))

PartialDependenceDisplay.from_estimator(

    model, X_train, features,

    feature_names=['Rainfall Intensity (P)', 'Slope', 'T/Tc'],

    ax=ax

)

plt.suptitle('Partial Dependence Plots')

plt.tight_layout()

plt.show()

# %%

# Individual PDP plots with more details

fig, ax = plt.subplots(1, 3, figsize=(18, 5))

# PDP for Rainfall Intensity

PartialDependenceDisplay.from_estimator(

    model, X_train, [0],

    feature_names=['Rainfall Intensity (P)', 'Slope', 'T/Tc'],

    ax=ax[0]

)

ax[0].set_title('Partial Dependence on Rainfall Intensity')

ax[0].set_ylabel('Partial Dependence')

# PDP for Slope

PartialDependenceDisplay.from_estimator(

    model, X_train, [1],

    feature_names=['Rainfall Intensity (P)', 'Slope', 'T/Tc'],

    ax=ax[1]

)

ax[1].set_title('Partial Dependence on Slope')

# PDP for T/Tc

PartialDependenceDisplay.from_estimator(

    model, X_train, [2],

    feature_names=['Rainfall Intensity (P)', 'Slope', 'T/Tc'],

    ax=ax[2]

)

ax[2].set_title('Partial Dependence on T/Tc')

plt.tight_layout()

plt.show()

# %%

# 2D PDP plot for interactions between features

print("\nCreating 2D PDP plots to show feature interactions...")

# Rainfall Intensity vs Slope

fig, ax = plt.subplots(figsize=(8, 6))

PartialDependenceDisplay.from_estimator(

    model, X_train, [(0, 1)],

    feature_names=['Rainfall Intensity (P)', 'Slope', 'T/Tc'],

    ax=ax

)

plt.title('Partial Dependence: Rainfall Intensity vs Slope')

plt.tight_layout()

plt.show()

# Rainfall Intensity vs T/Tc

fig, ax = plt.subplots(figsize=(8, 6))

PartialDependenceDisplay.from_estimator(

    model, X_train, [(0, 2)],

    feature_names=['Rainfall Intensity (P)', 'Slope', 'T/Tc'],

    ax=ax

)

plt.title('Partial Dependence: Rainfall Intensity vs T/Tc')

plt.tight_layout()

plt.show()

# Slope vs T/Tc

fig, ax = plt.subplots(figsize=(8, 6))

PartialDependenceDisplay.from_estimator(

    model, X_train, [(1, 2)],

    feature_names=['Rainfall Intensity (P)', 'Slope', 'T/Tc'],

    ax=ax

)

plt.title('Partial Dependence: Slope vs T/Tc')

plt.tight_layout()

plt.show()

# %%

# Interactive PDP plots using plotly (optional)

try:

    from sklearn.inspection import partial_dependence

    import plotly.graph_objects as go

    print("\nCreating interactive PDP plots...")

    # Create PDP data

    pdp_Rainfall = partial_dependence(model, X_train, [0])

    pdp_Slope = partial_dependence(model, X_train, [1])

    pdp_TTc = partial_dependence(model, X_train, [2])

    # Create figures

    fig1 = go.Figure()

    fig1.add_trace(go.Scatter(x=pdp_Rainfall['values'][0], y=pdp_Rainfall['average'][0],

                             mode='lines+markers', name='Rainfall Intensity'))

    fig1.update_layout(title='Partial Dependence on Rainfall Intensity (P)',

                      xaxis_title='Rainfall Intensity (P)',

                      yaxis_title='Partial Dependence')

    fig2 = go.Figure()

    fig2.add_trace(go.Scatter(x=pdp_Slope['values'][0], y=pdp_Slope['average'][0],

                             mode='lines+markers', name='Slope'))

    fig2.update_layout(title='Partial Dependence on Slope',

                      xaxis_title='Slope',

                      yaxis_title='Partial Dependence')

    fig3 = go.Figure()

    fig3.add_trace(go.Scatter(x=pdp_TTc['values'][0], y=pdp_TTc['average'][0],

                             mode='lines+markers', name='T/Tc'))

    fig3.update_layout(title='Partial Dependence on T/Tc',

                      xaxis_title='T/Tc',

                      yaxis_title='Partial Dependence')

    fig1.show()

    fig2.show()

    fig3.show()

except ImportError:

    print("Plotly not installed. Skipping interactive plots. Install with: !pip install plotly")

# %%

# Feature importance analysis

print("\nFeature importance from the Random Forest model:")

importances = model.feature_importances_

feature_names = X.columns

# Sort feature importances

indices = np.argsort(importances)[::-1]

# Plot

plt.figure(figsize=(10, 5))

plt.title("Feature Importances")

plt.bar(range(X.shape[1]), importances[indices], align="center")

plt.xticks(range(X.shape[1]), [feature_names[i] for i in indices])

plt.xlabel("Features")

plt.ylabel("Importance Score")

plt.show()

# %%

# Print numerical importance values

print("\nNumerical feature importance values:")

for i, (feature, importance) in enumerate(zip(feature_names[indices], importances[indices])):

    print(f"{i+1}. {feature}: {importance:.4f}")

Permutation Features Importance Code

# 1. Import necessary libraries

import numpy as np

import pandas as pd

import matplotlib.pyplot as plt

from sklearn.ensemble import RandomForestRegressor

from sklearn.inspection import permutation_importance

from sklearn.model_selection import train_test_split

# 2. Upload and read your dataset

from google.colab import files

uploaded = files.upload()

# 3. Load your data

data = pd.read_excel(list(uploaded.keys())[0])

# 4. Define independent variables (X) and dependent variable (y)

X = data[['Rainfall Intensity (P)', 'Slope ', 'T/Tc']]

y = data['Q']

# 5. Split the dataset into training and testing

X_train, X_test, y_train, y_test = train_test_split(X, y, test_size=0.2, random_state=42)

# 6. Train Random Forest model

rf = RandomForestRegressor(n_estimators=100, random_state=42)

rf.fit(X_train, y_train)

# 7. Perform Permutation Importance

result = permutation_importance(rf, X_test, y_test, n_repeats=30, random_state=42)

# 8. Create a DataFrame for importances

importance_df = pd.DataFrame({

    'Feature': X.columns,

    'Importance Mean': result.importances_mean,

    'Importance Std': result.importances_std

})

# 9. Sort by Importance

importance_df = importance_df.sort_values(by='Importance Mean', ascending=True)

# 10. Plot the Permutation Importance

plt.figure(figsize=(8,6))

# Draw horizontal bar plot

plt.barh(

    y=importance_df['Feature'],

    width=importance_df['Importance Mean'],

    xerr=importance_df['Importance Std'],

    color='skyblue',

    edgecolor='black',

    height=0.4   # Reduced bar thickness

)

# Titles and labels with Times New Roman font

plt.title('Permutation Feature Importance (RF Model)', fontsize=14, fontname='Times New Roman')

plt.xlabel('Importance Mean', fontsize=12, fontname='Times New Roman')

plt.ylabel('Feature', fontsize=12, fontname='Times New Roman')

# Remove gridlines

plt.grid(False)

# Adjust layout

plt.tight_layout()

# Show plot

plt.show()

K-Folds Cross-Validation Code

# %% [markdown]

"""

# Random Forest with 10-Fold Cross-Validation & Excel Export

This notebook:

1. Trains Random Forest with 10-fold CV

2. Calculates R², RMSE, MAE, NSE, KGE

3. Exports results to Excel

"""

# %%

# Install necessary libraries

!pip install pandas numpy matplotlib scikit-learn openpyxl

# %%

import pandas as pd

import numpy as np

from sklearn.ensemble import RandomForestRegressor

from sklearn.model_selection import KFold

from sklearn.metrics import mean_squared_error, mean_absolute_error, r2_score

from sklearn.preprocessing import StandardScaler

from google.colab import files

# Custom metric functions

def nash_sutcliffe(y_true, y_pred):

    return 1 - (np.sum((y_true - y_pred)**2) / np.sum((y_true - np.mean(y_true))**2))

def kling_gupta(y_true, y_pred):

    r = np.corrcoef(y_true, y_pred)[0,1]

    alpha = np.std(y_pred) / np.std(y_true)

    beta = np.mean(y_pred) / np.mean(y_true)

    return 1 - np.sqrt((r-1)**2 + (alpha-1)**2 + (beta-1)**2)

# %%

# Upload the Excel file manually

uploaded = files.upload()

# %%

# Read the uploaded file

file_name = list(uploaded.keys())[0]

df = pd.read_excel(file_name)

df.columns = df.columns.str.strip()  # Clean column names

# Features and Target

X = df[['Rainfall Intensity (P)', 'Slope', 'T/Tc']]

y = df['Q']

# Random Forest doesn't require feature scaling, but we'll keep it for consistency

scaler = StandardScaler()

X_scaled = scaler.fit_transform(X)

# %%

# 10-Fold Cross-Validation

print("Running 10-fold cross-validation for Random Forest...")

kf = KFold(n_splits=10, shuffle=True, random_state=42)

results = []

for fold, (train_idx, test_idx) in enumerate(kf.split(X_scaled)):

    X_train, X_test = X_scaled[train_idx], X_scaled[test_idx]

    y_train, y_test = y.iloc[train_idx], y.iloc[test_idx]

    model = RandomForestRegressor(

        n_estimators=100,

        max_depth=None,

        min_samples_split=2,

        random_state=42

    )

    model.fit(X_train, y_train)

    y_pred = model.predict(X_test)

    metrics = {

        'Fold': fold+1,

        'R²': r2_score(y_test, y_pred),

        'RMSE': np.sqrt(mean_squared_error(y_test, y_pred)),

        'MAE': mean_absolute_error(y_test, y_pred),

        'NSE': nash_sutcliffe(y_test, y_pred),

        'KGE': kling_gupta(y_test, y_pred)

    }

    results.append(metrics)

    print(f"Fold {fold+1} completed")

# Create DataFrame from results

results_df = pd.DataFrame(results)

# Calculate averages

avg_metrics = {

    'Fold': 'Average',

    'R²': results_df['R²'].mean(),

    'RMSE': results_df['RMSE'].mean(),

    'MAE': results_df['MAE'].mean(),

    'NSE': results_df['NSE'].mean(),

    'KGE': results_df['KGE'].mean()

}

# Append averages to results

results_df = pd.concat([results_df, pd.DataFrame([avg_metrics])], ignore_index=True)

# %%

# Export to Excel

output_file = "RandomForest_CV_Results.xlsx"

with pd.ExcelWriter(output_file) as writer:

    # Main results sheet

    results_df.to_excel(writer, sheet_name='CV Results', index=False)

    # Summary statistics sheet

    summary_stats = results_df.iloc[:-1].describe().loc[['mean', 'std', 'min', 'max']]

    summary_stats.to_excel(writer, sheet_name='Summary Statistics')

    # Model info sheet

    model_info = pd.DataFrame({

        'Parameter': ['Model', 'n_estimators', 'max_depth',

                     'min_samples_split', 'random_state', 'CV Folds'],

        'Value': ['RandomForest', 100, 'None', 2, 42, 10]

    })

    model_info.to_excel(writer, sheet_name='Model Info', index=False)

# Download the file

files.download(output_file)

print(f"\nResults exported to {output_file}")

# Display final results

print("\nFinal Results:")

display(results_df)

# Feature Importance (Bonus)

feature_importance = pd.DataFrame({

    'Feature': df[['Rainfall Intensity (P)', 'Slope', 'T/Tc']].columns,

    'Importance': model.feature_importances_

}).sort_values('Importance', ascending=False)

print("\nFeature Importance:")

display(feature_importance)

Artificial Model Based Prediction Code

# Install required packages

!pip install openpyxl scikit-learn matplotlib seaborn

# Import necessary libraries

import pandas as pd

import numpy as np

import matplotlib.pyplot as plt

import seaborn as sns

from sklearn.ensemble import RandomForestRegressor, VotingRegressor

from sklearn.svm import SVR

from sklearn.model_selection import train_test_split

from sklearn.preprocessing import StandardScaler

from sklearn.metrics import mean_squared_error, r2_score

from google.colab import files

# Upload the Excel file

uploaded = files.upload()

# Read the Excel file

file_name = next(iter(uploaded))

df = pd.read_excel(file_name, engine='openpyxl')

# Display original column names

print("Original column names:")

print(df.columns.tolist())

# Clean column names: strip whitespace and convert to lowercase

df.columns = df.columns.str.strip().str.lower()

# Display cleaned column names

print("\nCleaned column names:")

print(df.columns.tolist())

# Rename columns for consistency

df.rename(columns={

    'rainfall intensity (p)': 'P',

    'slope   (%)': 'slope',

    't/tc': 'T_Tc',

    'q': 'Q'

}, inplace=True)

# Display renamed column names

print("\nRenamed column names:")

print(df.columns.tolist())

# Define features and target variable

X = df[['T_Tc', 'slope', 'P']]

y = df['Q']

# Split the dataset into training and testing sets (80% train, 20% test)

X_train, X_test, y_train, y_test = train_test_split(X, y, test_size=0.2, random_state=42)

# Feature scaling

scaler = StandardScaler()

X_train_scaled = scaler.fit_transform(X_train)

X_test_scaled = scaler.transform(X_test)

# Initialize individual models

rf = RandomForestRegressor(n_estimators=100, random_state=42)

svr = SVR(kernel='rbf', C=100, gamma=0.1, epsilon=0.1)

# Create a Voting Regressor ensemble

voting_reg = VotingRegressor(estimators=[('rf', rf), ('svr', svr)])

# Train the ensemble model

voting_reg.fit(X_train_scaled, y_train)

# Predict on the test set

y_pred = voting_reg.predict(X_test_scaled)

# Evaluate the model

rmse = np.sqrt(mean_squared_error(y_test, y_pred))

r2 = r2_score(y_test, y_pred)

print(f"\nRoot Mean Squared Error (RMSE): {rmse:.2f}")

print(f"R-squared (R²): {r2:.2f}")

# Plot Actual vs Predicted values

plt.figure(figsize=(8, 6))

plt.scatter(y_test, y_pred, color='blue', edgecolor='k', alpha=0.7)

plt.plot([y_test.min(), y_test.max()], [y_test.min(), y_test.max()], 'r--', lw=2)

plt.xlabel('Actual Peak Discharge (Q)')

plt.ylabel('Predicted Peak Discharge (Q)')

plt.title('Actual vs Predicted Peak Discharge')

plt.grid(True)

plt.tight_layout()

plt.show()
